# Application of IVDr NMR spectroscopy to stratify Parkinson’s disease with absolute quantitation of blood serum metabolites and lipoproteins

**DOI:** 10.1038/s41598-025-01352-0

**Published:** 2025-05-22

**Authors:** Georgy Berezhnoy, Gyuntae Bae, Leonie Wüst, Claudia Schulte, Claire Cannet, Isabel Wurster, Milan Zimmermann, Alexander Jäck, Eike Jakob Spruth, Julian Hellmann-Regen, Sandra Roeske, Dominik Pürner, Wenzel Glanz, Fabian Maass, Felix Hufschmidt, Ingo Kilimann, Elisabeth Dinter, Okka Kimmich, Anna Gamez, Johannes Levin, Josef Priller, Oliver Peters, Michael Wagner, Alexander Storch, Paul Lingor, Emrah Düzel, Christoph van Riesen, Ullrich Wüllner, Stefan Teipel, Björn Falkenburger, Mathias Bähr, Inga Zerr, Gabor C. Petzold, Annika Spottke, Patricia Rizzu, Frederic Brosseron, Hartmut Schäfer, Thomas Gasser, Christoph Trautwein

**Affiliations:** 1 Werner Siemens Imaging Center, Department of Preclinical Imaging and Radiopharmacy, University of Tübingen, Tübingen, Germany; 2 Hertie Institute for Clinical Brain Research, Department of Neurodegenerative Diseases, University of Tübingen, Tübingen, Germany; 3 German Center for Neurodegenerative Diseases (DZNE), Tübingen, Germany; 4 Bruker BioSpin GmbH & Co. KG (AIC Division), Ettlingen, Germany; 5 German Center for Neurodegenerative Diseases (DZNE), Munich, Germany; 6 Department of Neurology, University Hospital of Munich, Ludwig-Maximilians-Universität (LMU) Munich, Munich, Germany; 7 German Center for Neurodegenerative Diseases (DZNE), Berlin, Germany; 8 Neuropsychiatry and Laboratory of Molecular Psychiatry, Department of Psychiatry and Psychotherapy, Charité - Universitätsmedizin Berlin, Berlin, Germany; 9 Department of Psychiatry and Neurosciences, Charité Universitätsmedizin Berlin, Berlin, Germany; 10 ECRC Experimental and Clinical Research Center, Charité – Universitätsmedizin Berlin, Berlin, Germany; 11 German Center for Neurodegenerative Diseases (DZNE), Bonn, Germany; 12 Department of Neurology, School of Medicine, University Hospital München rechts der Isar, Technical University of Munich, Munich, Germany; 13 German Center for Neurodegenerative Diseases (DZNE), Magdeburg, Germany; 14 Institute of Cognitive Neurology and Dementia Research, Otto-von-Guericke University, Magdeburg, Germany; 15 Clinic for Neurology, Medical Faculty, University Hospital Magdeburg, Magdeburg, Germany; 16 Department of Neurology, University Medical Center, Georg August University, Göttingen, Germany; 17 Department of Old Age Psychiatry and Cognitive Disorders, University Hospital Bonn and University of Bonn, Bonn, Germany; 18 German Center for Neurodegenerative Diseases (DZNE), Rostock-Greifswald, Germany; 19 Department of Psychosomatic Medicine, Rostock University Medical Center, Rostock, Germany; 20 German Center for Neurodegenerative Diseases (DZNE), Dresden, Germany; 21 Department of Neurology, University Hospital Carl Gustav Carus, Technische Universität Dresden, Dresden, Germany; 22 Department of Vascular Neurology, University Hospital Bonn, Bonn, Germany; 23 Munich Cluster for Systems Neurology (SyNergy) Munich, Munich, Germany; 24 Department of Psychiatry and Psychotherapy, School of Medicine and Health, Technical University of Munich, German Center for Mental Health (DZPG), Munich, Germany; 25 University of Edinburgh and UK DRI, Edinburgh, UK; 26 Charité – Universitätsmedizin Berlin, corporate member of Freie Universität Berlin and Humboldt-Universität zu Berlin-Institute of Psychiatry and Psychotherapy, Berlin, Germany; 27 Department of Neurology, University Medical Centre, Rostock, Germany; 28 Institute of Cognitive Neuroscience, University College London, London, UK; 29 German Center for Neurodegenerative Diseases (DZNE), Goettingen, Germany; 30 Department of Neurology, University of Bonn, Bonn, Germany; 31 M3 Research Center for Malignome, Metabolome and Microbiome, Medical Faculty, University of Tübingen, Tübingen, Germany; 32 Core Facility Metabolomics, Medical Faculty, University of Tübingen, Tübingen, Germany

**Keywords:** Parkinson’s disease, *GBA*, Recessive inheritance, Blood, Dementia, Biomarkers, Neurological disorders, Biochemistry, Neuroscience, Metabolomics

## Abstract

The challenge of early detection and stratification in Parkinson’s disease (PD) is urgent due to the current emergence of mechanism-based disease-modifying treatments. In here, metabolomic and lipidomic parameters obtained by a standardized and targeted in vitro diagnostic research (IVDr) platform have a significant potential to address therapy-related questions and generate improved biomarker panels. Our study aimed to use IVDr nuclear magnetic resonance (NMR) spectroscopy to quantify metabolites and lipoproteins in PD blood serum from different cohorts to stratify metabolically driven subtypes of idiopathic and genetic PD. Serum aliquots from three neurodegeneration biobank cohorts (287 samples in total, including 62 PD patient samples with GBA mutation, 98/43 PD patient samples of early/late stages of disease duration, 20 PD samples from patients with mutations in recessive PD genes and some smaller subgroups of mitochondrial and double mutation cases) were prepared and analyzed with IVDr NMR spectroscopy, covering 39 blood serum metabolites and 112 lipoprotein parameters. Uni- and multivariate statistics were used to identify metabolism-driven changes under consideration of typical confounders such as age, sex and disease duration and set into context with clinical biomarkers such as CSF concentrations of alpha-synuclein, neurofilament light chain, and tau protein. Based on the different PD subgroups we performed a total of eight different comparisons. Highlights from these comparisons include increased citrate and dimethylglycine with a decrease of creatinine and methionine in healthy controls and early PD group compared to GBA, PD late and recessive PD. We furthermore identified decreased HDL-3 free cholesterol in genetic PD cases compared to sporadic subject samples (sum of the PD early and PD late groups). Considering medication, we found that the levodopa equivalent daily dose (LEDD) is mostly positively correlated with tyrosine and citrate in sporadic PD compared to pyruvate and phenylalanine in genetic PD. Cerebrospinal fluid levels of alpha-synuclein were negatively correlated with alanine. Further metabolites and lipoproteins with discriminatory power for double mutation PD cases involved ornithine, 2-aminobutyrate and 2-hydroxybutyrate as well as for mitochondrial phenotypes via LDL phospholipid, apolipoprotein and cholesterol subfractions. Quantitative IVDr NMR serum spectroscopy is able to stratify PD patient samples of different etiology and can contribute to a wider understanding of the underlying metabolism-driven alterations e.g. in energy, amino acid, and lipoprotein metabolism. Though our overall cohort was large, major confounders such as age, sex and medication have a strong impact. That is why absolute quantification and detailed patient knowledge about metabolic confounders, is a premise for future translation of NMR serum spectroscopy to routine PD diagnostics.

**Supplementary Information:**

The online version contains supplementary material available at 10.1038/s41598-025-01352-0.

## Introduction

Parkinson’s disease (PD) is characterized by a unique combination of motor and non-motor symptoms^1^. While preclinical PD models suggest new targets for therapy^2^, there is huge lack to develop new robust biomarkers for early phases of the disease^3^.

In recent years, omics techniques (genomics, proteomics, transcriptomics, and metabolomics) have entered the research field to study PD pathology and pathogenesis^4^. From the genetic standpoint, the beta-glucocerebrosidase gene (*GBA*) mutation, also causing Gaucher’s disease, is a critical genetic variable^5^, which needs to be considered in population-wide studies of PD. In a recent study^6^, the increasing importance of proteomics and transcriptomics was further demonstrated in network-building (including correlations between miRNAs and proteins) and highlighting the potential PD biomarker *14-3-3 protein zeta/delta* (YWHAZ) protein. Overall, cooperation of omics methods of PD population screening (genomics – via genome-wide association studies (GWAS), transcriptomics, and proteomics) tend to give complimentary results and allow to investigate processes behind protein folding (aggregations)^7^.

Metabolomics research in PD has proven to help screen tissue or biofluid samples for low molecular mass compounds with polar and lipid characteristics^8^. Metabolomic studies can provide essential insights into pathologic alterations of metabolic pathways and may lead to discovering new PD biomarkers. Using metabolomics, two analytical techniques are used to study PD: mass spectrometry (MS) and nuclear magnetic resonance (NMR) spectroscopy^9,10^. Unlike MS, NMR based metabolomics offers the benefit of a non-destructive sample analysis and precise absolute quantification^11,12^. On the other hand, NMR suffers from poor sensitivity and metabolites can only be identified at relatively high concentrations. For any metabolomics investigation, the acquiring large sets of patient material with associated metadata is essential to contribute to a better understanding of Parkinson’s disease.

The utilization of NMR-based metabolic and lipoprotein parameters in combination with clinical biomarkers for PD, neurodegeneration and aging has been previously used in PD research^13,14^ and such combinations may reach a certain level of stratification in a PD phenotype, as was shown in a study by Troisi et al. with the use of gas chromatography - mass spectrometry^15^. One major research field is to identify significant metabolic anomalies that allow to distinguish iPD from other PD types.

In our labs, we recently applied quantitative NMR spectroscopy to examine serum and CSF samples from AD (Alzheimer´s disease) and MCI (mild cognitive impairment, investigated with AD biomarkers in CSF) patients^16,17^. Thus, the aim of our study was to elaborate how the NMR-based analysis of blood serum and lipoprotein parameters of different PD subtypes could be linked to genotypes and existing clinical indicators to expand the diagnostic PD toolbox. Such investigations may, at some point in the future, lead to novel screening programs or improved patient care e.g. using this technology as monitoring tool within curative approaches^18^.

## Results

### Analysis 1: the comparison of main PD groups elucidates imbalances in blood metabolites

**Fig. 1:**
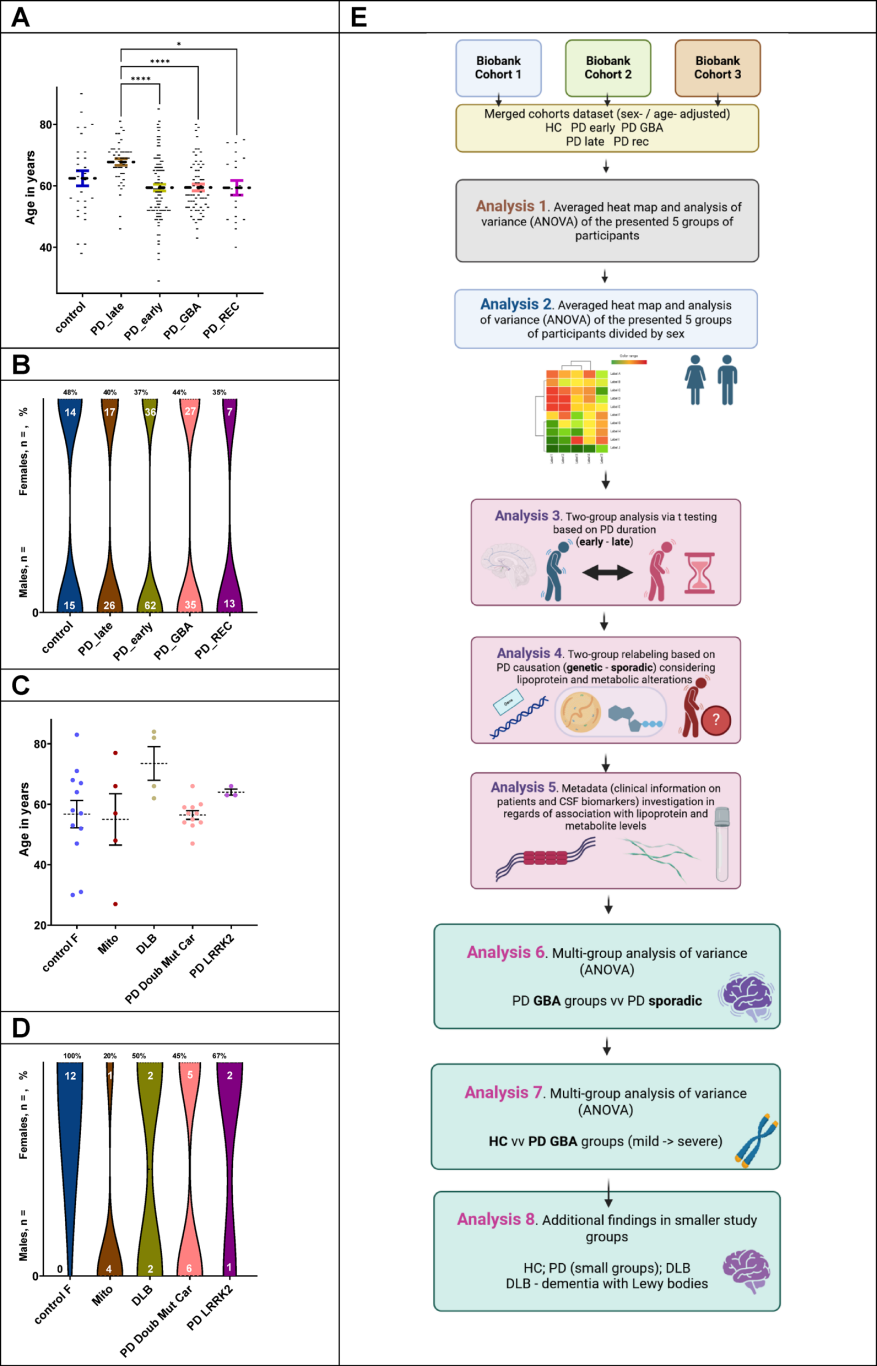
Overview of the cohorts, sample numbers and plan of study. (**A**) Descriptive statistics plot (mean ± SEM, SEM - Standard Error of the Mean) on age, showing the significantly higher age of the Sporadic PD late group (main 5 groups); (**B**) Descriptive statistics plot on sex in which a balance corridor of 40% ±10% among groups is observed (main 5 groups); (**C**) Descriptive statistics plot (mean ± SEM, SEM - Standard Error of the Mean) on age (remaining groups); (**D**) Descriptive statistics plot on sex (remaining groups); (**E**) Overall study plan– Created in BioRender. Berezhnoy, G. (2025) https://BioRender.com/m96r807.

**Fig. 2:**
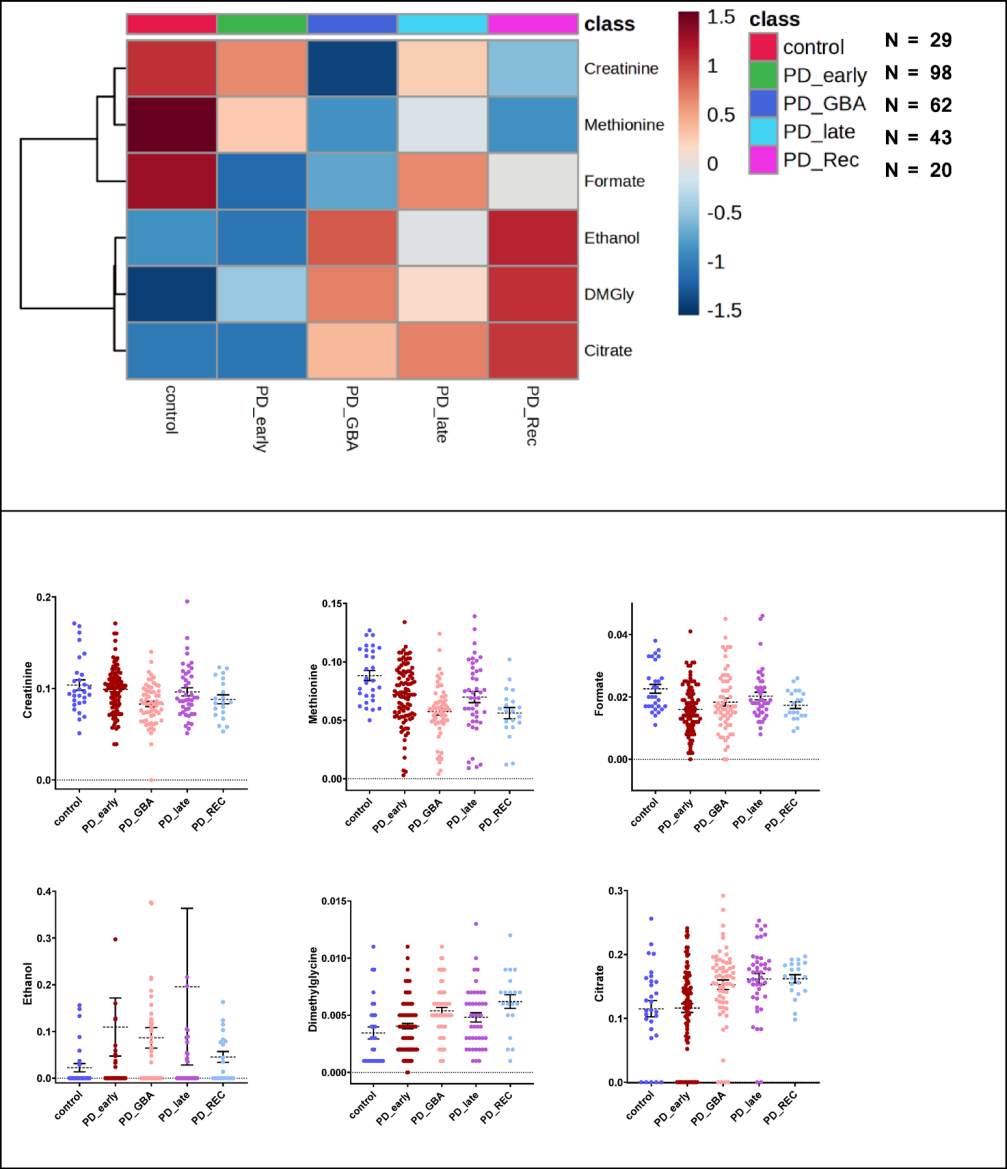
Significant metabolite alterations within the five main studied groups. Heatmap of FDR significant findings checked via ANOVA (*p* < 0.05). The six significantly changed metabolites are creatinine, methionine, formate, ethanol, dimethylglycine (DMGly) and citrate (shown as dot plots, mean ± SEM). For Ethanol plot 9 dots were not plotted.

The overall cohort is the sum of three subproject cohorts that were measured at different time spans between 2021 and 2023 and whose samples derived from two different biobanks, as illustrated in Table 1; Figs. 1 and 2. Serum samples for cohorts 1 and 3 were provided by a local biobank in Tübingen, Germany, whereas the subproject cohort 2 samples derived from a biobank at the DZNE in Bonn, Germany. The pooling of these cohorts allowed to reach higher n-numbers which are mandatory for a metabolomics approach.

**Table 1:** Overview of the analyzed overall and subproject cohorts (panel A) and the demographic and clinical metadata (panel B).

A			
Subproject Cohort 1 (2021)	Subproject Cohort 2 (2023)	Subproject Cohort 3 (2023)	Total Cohort (2021–2023)
(PD_*GBA*) *n* = 18; 18 *GBA* severe	(PD_*GBA*) *n* = 6; 1 *GBA* mild; 3 *GBA* risk; 1 *GBA* severe; 1 *GBA* VUS	(PD_*GBA*) *n* = 38; 12 *GBA* mild; 18 *GBA* risk; 4 *GBA* severe; 4 *GBA* VUS	(PD_*GBA*) *n* = 62; 13 *GBA* mild; 21 *GBA* risk; 23 *GBA* severe; 5 *GBA* VUS
(Control) *n* = 13	(Control) *n* = 16	–	(Control) *n* = 29
(Control_F) *n* = 8; female surplus group	(Control_F) *n* = 4; female surplus group	–	(Control_F) *n* = 12; female surplus group
(Mitochon. dis.) *n* = 5; 1 Kearns-Sayre Syndrome; 1 PD & mitochondriopathy; 1 schizophrenia & mitochondriopathy; 1 sarcoidosis	–	–	(Mitochon. dis.) *n* = 5
–	(DLB) *n* = 4	–	(DLB) *n* = 4
(Sporadic PD early) *n* = 20	(Sporadic PD early) *n* = 36	(Sporadic PD early) *n* = 42	(Sporadic PD early) *n* = 98
(Sporadic PD late) *n* = 18	(Sporadic PD late) *n* = 10	(Sporadic PD late) *n* = 15	(Sporadic PD late) *n* = 43
(PD rec) *n* = 17; 1 *PINK1* biA; 1 *PINK1* monoA; 4 *PRKN* biA; 11 *PRKN* monoA	–	(PD rec) *n* = 3; 3 PD *PRKN* monoA	(PD rec) *n* = 20
(PD double mutation carrier) *n* = 4; 2 PD *GBA PRKN* monoA; 1 PD *GBA PARK7* monoA; 1 PD *GBA LRRK2*	(PD double mutation carrier) *n* = 2; 2 PD *GBA LRRK2*	(PD double mutation carrier) *n* = 5; 1 PD *GBA PARK7*; 1 PD *GBA LRRK2*; 3 PD *GBA PRKN* biA	(PD double mutation carrier) *n* = 11
(PD *LRRK2*) *n* = 1	(PD *LRRK2*) *n* = 1	(PD *LRRK2*) *n* = 1	(PD *LRRK2*) *n* = 3
**B**			
**Demographic data (Total)**		**Clinical data (Total)**	
Sex (women) PD_*GBA* Control (matched) Sporadic PD early Sporadic PD late PD rec	Women (number; %) 27; *43.5%* 14; *48.3%* 36; *36.7%* 17; *39.5%* 7; *35.0%*	Age PD onset (y.o., mean ± SD) PD_*GBA* Control Sporadic PD early Sporadic PD late PD rec	Age PD onset (years) 52 ± 9 N/A 57 ± 11 56 ± 7 47.5 ± 12
Age (y.o., mean ± SD) PD_*GBA* Control (matched) Sporadic PD early Sporadic PD late PD rec	Age (years) 59 ± 9 62 ± 13 59 ± 11 68 ± 7 59 ± 11	PD disease duration (years, mean ± SD) PD_*GBA* Control Sporadic PD early Sporadic PD late PD rec	PD disease duration (years) 7.5 ± 5 0 ± 0 3 ± 1 11 ± 3 12 ± 10

PD_*GBA* – PD diagnosed patients (samples) with confirmed mutations in the gene for glucocerebrosidase (*GBA*); VUS – variants of unknown significance; Control – non-parkinsonian controls; Control_F – excluded subset of female participants due to age and&nbsp;sex matching of other groups of the study; Sporadic PD early – patients (samples) screened with diagnosed PD prior 5 or less years since the age of onset; Sporadic PD late – patients (samples) screened with diagnosed PD prior 6 or more years since the age of onset; PD rec – confirmed recessive forms of PD; A – allelic; DLB – dementia with Lewy Bodies. The means were calculated as averages. SD – standard deviation (st dev). n – number of samples.

Full clinical metadata and CSF biomarkers are provided in Supplementary Table 1. The following ID numbers and attributes have been used within the Supplementary Table 1. The *GBA* genetic variations have been labeled additionally with the grade of Gaucher’s disease severity and their positive association with PD^19,20^ and is increasing as follows: *GBA* risk → *GBA* mild → *GBA* severe. VUS label means “variance of unclear significance”.

Our present study classified patients as having either sporadic PD early or sporadic PD late. Those with PD duration parameter within five years were classified as having sporadic PD early entry, while those with more than five years were classified as having sporadic PD late group label.

The supplementary material also includes entries for comorbidities in non-PD controls (from all three cohorts of the current study). Five individuals, in our investigation, are marked as “Mito” because these patients were born with recessive mitochondrial diseases. It is also important to highlight another small group of participants diagnosed with dementia with Lewy bodies (DLB). The DLB condition was in included in the sporadic analysis group.

Comparing the 5 main groups (Analysis 1 in Fig. 1E), methionine had a significant decrease (FDR < 0.05) in recessive PD, *GBA*, and late PD patient groups (Fig. 2). The non-wildtype PD groups (PD recessive, *GBA*) displayed lower creatinine levels and higher dimethylglycine (DMGly). Further significant metabolites were higher citric acid (citrate) levels in late and recessive PD and ethanol, which had many missing values (below limit of detection) as displayed in the violin plot. We observed that the highest blood formate levels were found in the group of control samples. After testing for FDR significant ANOVA variables in these five main patient groups, we could locate covariant dependencies between the main checked factors (PD disease duration, age, and sex) and ethanol (Supplementary Table 2).

### Analysis 2: male PD subjects are characterized by higher levels of VLDL and valine while females show increased LDL and HDL lipoproteins

Performing Analysis 2 in Fig. 1E and applying a stricter FDR-adjustment (*p* < 0.001, Supplementary Fig. 2) filter, numerous variables changed, when separately analyzing male and female subgroups (using Ward clustering method). Doing so, we spotted in males (but interestingly also PD recessive females, Table 1) higher levels of very low-density lipoprotein (VLDL) parameters. Males on average had higher blood creatinine and valine in comparison to females. Furthermore, the BCAAs leucine and isoleucine as well as Glyc/SPC were elevated in male cohorts. Finally, we identified citrate and DMGly being higher in some but not all male groups. In the subset of female groups, we found increased LDL subfractions LDL1-LDL3, creatine, HDL subfractions HDL1-HDL3, overall HDL content, and the SPC peak (supramolecular phospholipid composite) in comparison to males.

### Analysis 3: sporadic PD early and sporadic PD late patients can be distinguished by their blood citrate levels

Comparing NMR data of Sporadic PD early with Sporadic PD late (Analysis 3 in Fig. 1E) did not reveal any significantly changed lipoproteins; however, FDR-significantly changed citrate (Supplementary Fig. 2) in the Sporadic PD late group. We must note that the Sporadic PD late group is skewed toward higher patient ages, and therefore FDR significant results of citrate from the two-group t-test could be due to the aging factor. However, after analysis of covariance performance check, we found that citrate is not biased towards the PD disease duration variable, age, and sex; and its final covariate p value is significant (Supplementary Table 2).

It was determined, for our cohorts of patient samples, that citrate portrayed the strongest *t*-test significance result (with FDR-rate considered) at the level of p_FDR_ < 0.0125, while another TCA cycle-related metabolite, 2-oxoglutarate, was having p_FDR_ of ~ 0.125 reported value of discrimination between the PD (early) v (late) groups. Of course, when the FDR-adjustment is dropped, *t*-statistics becomes significant for both metabolites. Succinate, another TCA-related metabolite that is measurable by our analytical assay applied, was neither raw p-value-wise nor after an FDR-adjustment even considerable for noteworthy changes between the two patient samples groups.

### Analysis 4: genetic and sporadic PD patients are discriminated by HDL free cholesterol particles, creatinine, methionine and dimethylglycine

**Fig. 3:**
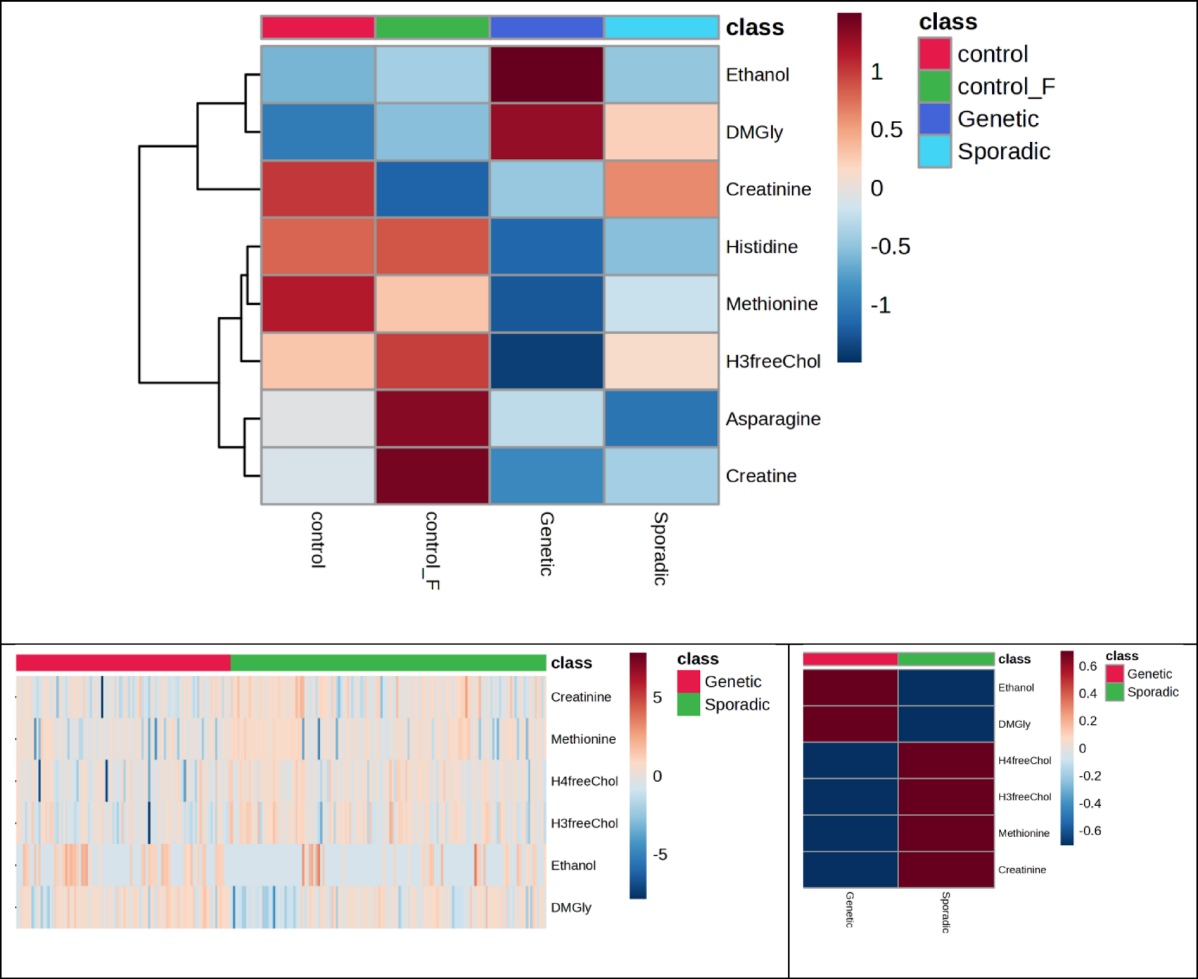
Significantly changed blood metabolites and lipoprotein comparing genetic with sporadic PD and control subject samples. Heatmap of FDR significant findings checked via ANOVA and t testing.

Comparing patient samples with sporadic (combination of PD early and late groups) against genetic PD (Analysis 4 in Fig. 1E, see Table 1, including double mutation carriers, PD recessive, PD *GBA*, and PD *LRRK2* individuals’ samples; *LRRK2* – leucine-rich repeat kinase 2) with two non-PD control groups (age- and sex-matched control group and group with subset of female participants due to age and sex matching; see Supplementary Table 2 for main statistics output), we were able to determine lowered histidine and methionine levels (Fig. 3).

Furthermore, creatinine was significantly lower in female controls and the genetic group. Of note, creatine was proven to be in covariance dependent on PD disease duration variable, or either to age or sex characteristics, therefore, the p value after covariance check is no longer significant, Supplementary Table 2). There were also technically variable changes of ethanol and asparagine (due to low n numbers). The HDL lipoprotein variable of HDL-3 free cholesterol was lowered in the PD genetic group, whereas dimethylglycine was elevated.

We also ran a significance t-test evaluation for only two diagnostic groups - genetic and sporadic PD. This observation of methionine and dimethylglycine was also proven to be FDR-significant (p_FDR_ = 0.006 – Methionine; p_FDR_ = 0.009 – DMGly). Overall, this additional two-group-based comparison highlighted six FDR-significant variables (Fig. 3), including two lipoprotein parameters from HDL subfractions of free cholesterol (HDL-3 and HDL-4). Characteristics of changes are the same also for the two-group comparison for those repeated parameters of the two analyses.

### Analysis 5: correlation of NMR parameters with CSF dementia biomarkers, α-synuclein, BMI index, Levodopa equivalent dosage daily, cognition and depression scores, and other relevant data

We additionally performed correlation analyses based on existing neurodegeneration biomarkers and clinical metadata (Analysis 5 in Fig. 1E). As not all metadata variables were available for all patients, the respective group sizes changed accordingly and the updated numbers are provided in the Methods section and Supplementary Table 3.

**Fig. 4:**
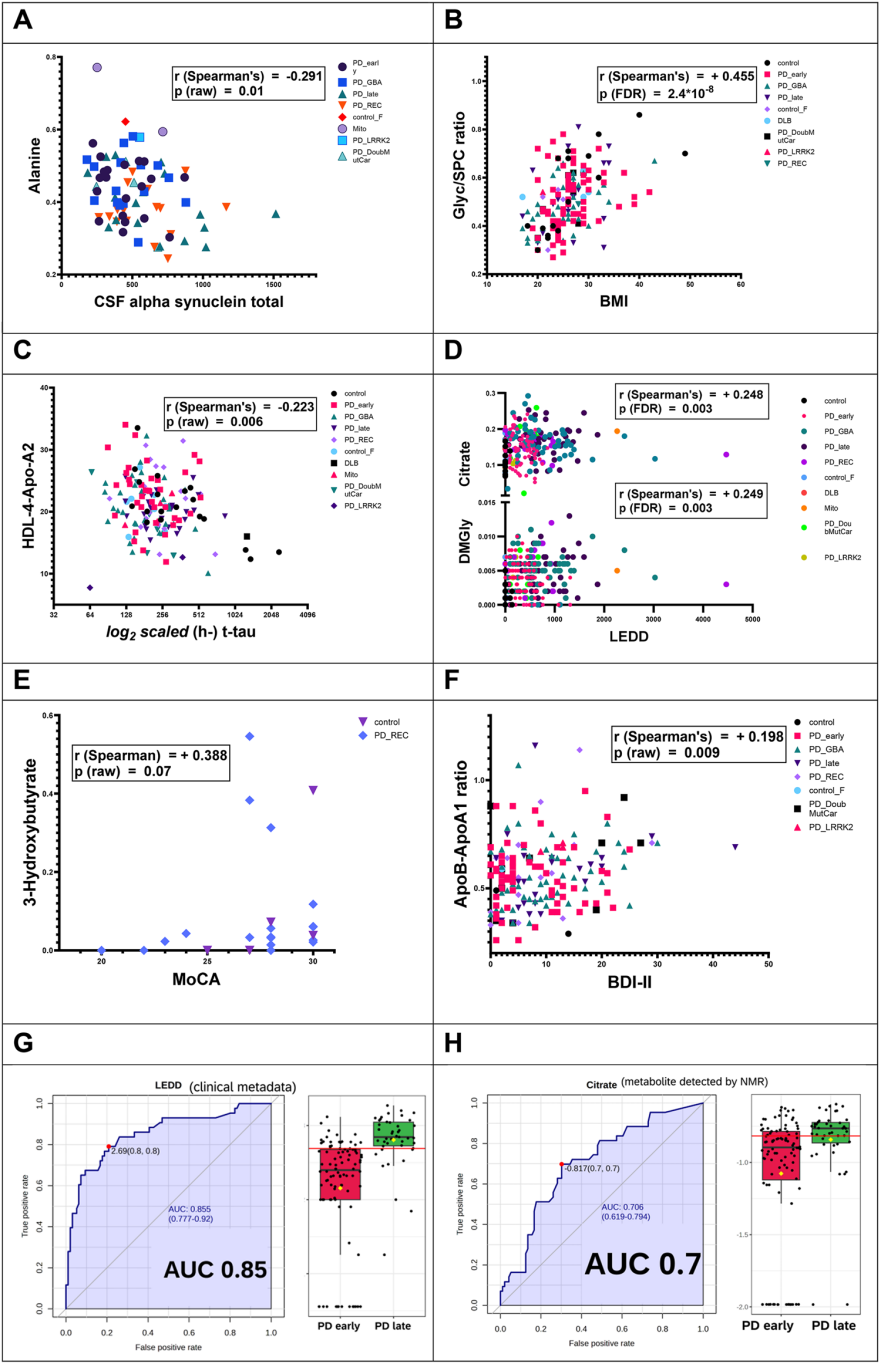

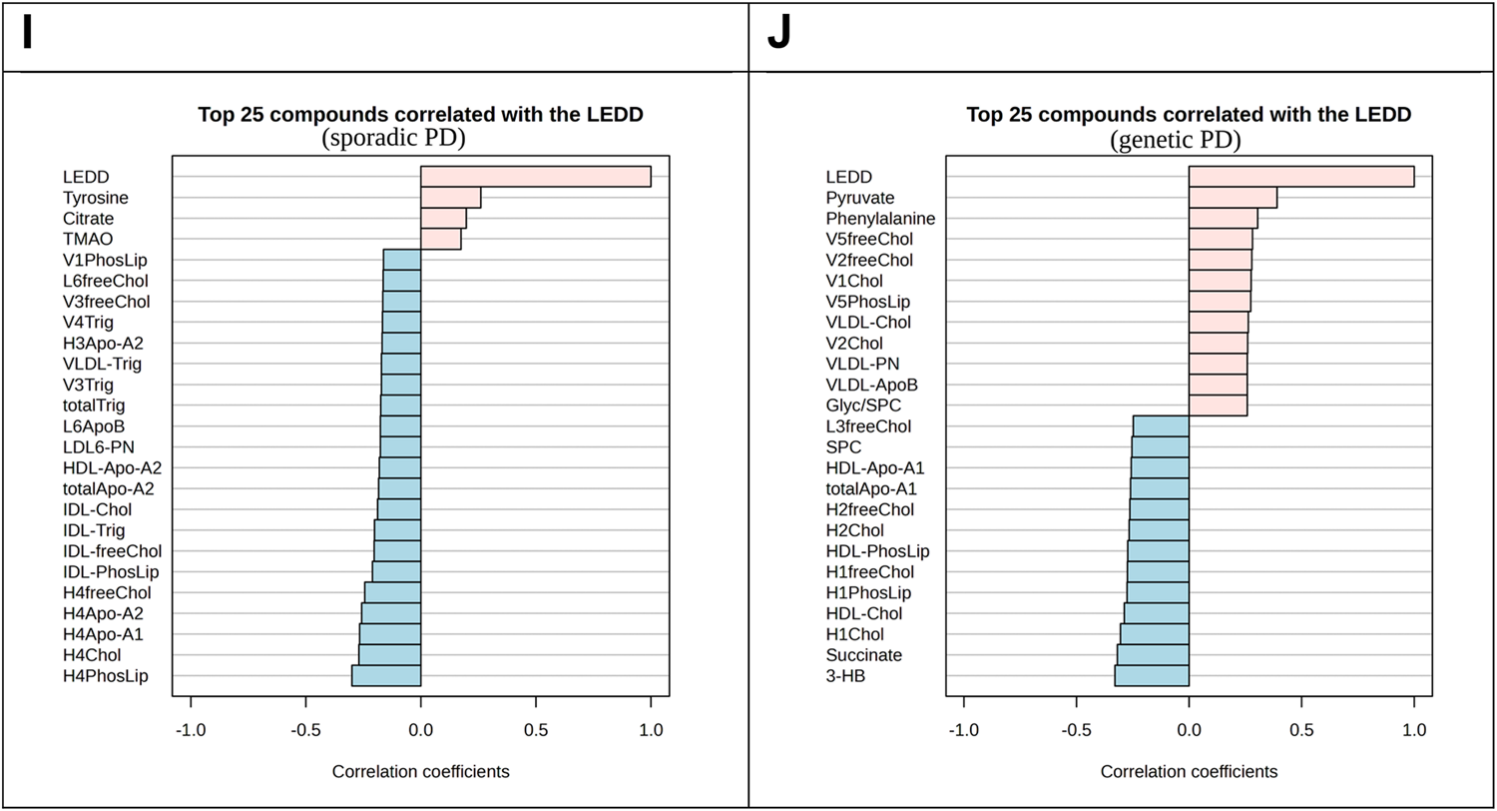
Correlations of clinical biomarkers and metadata with metabolites and lipoprotein alterations. (**A**) A negative correlation was found between blood alanine and CSF alpha-synuclein, (**B**) the Glyc/SPC ratio parameter shows a significant positive correlation with BMI index, (**C**) lipoprotein characteristic HDL-4 apolipoprotein Apo-A2 had a negative correlation with human total tau levels, (**D**) DMGly and citrate metabolites have positive correlations with LEDD, (**E**) blood 3-HB levels show a nonsignificant tendency to correlate to MoCA score (within non-PD Controls and PD recessive groups of individuals’ samples only), (**F**) blood ApoB100/ApoA1 ratio correlate positively with BDI-II, (**G**) area-under-curve analysis (receiver operating curve building) demonstrates a good statistical differentiation of Sporadic PD early and Sporadic PD late patient groups (n total = 139) for the LEDD available entries only, (**H**) area-under-curve analysis plot with a logarithmically normed box plot of blood citrate indicating of Sporadic PD early and Sporadic PD late patient groups (n total = 139) for the significant discriminatory characteristics tested separately based on the LEDD available entries only patient samples, (**I**) tyrosine shows a positive correlation with LEDD for PD sporadic (early, late; *n* = 139), (**J**) pyruvate has a positive correlation with LEDD for PD genetic groups (*GBA*, recessive, *LRRK2*, and double mutation carriers; *n* = 92). The software used places LEDD in the number one position, with a correlation of value plus one with itself (segments **I** and **J**). The rates shown on a (“classical”) Receiver Operating Characteristic (ROC) curve, often so, are also plotting the true positive rate (the sensitivity) against the false positive rate (1-specifcity) for different threshold settings of a diagnostic test, as performed in MetaboAnalyst^22^. The red separator line is representing an “optimal cutoff” that is according to located point furthest from the diagonal and according to the Youden’s method^23^.

Figure 4 and Supplementary Fig. 3 summarize our findings: First, we would like to highlight the negative correlation (*r* = −0.29, *p* = 0.01) between CSF total alpha-synuclein and blood alanine (which is lower in serum of late PD patients, when compared to Sporadic PD early). Second, the body mass index (BMI) was positively correlated with the inflammatory glycoprotein parameter Glyc/SPC ratio (FDR range of significance; *r* = 0.45, P_FDR_ < 10^−7^). Third, the correlation between HDL-4 apolipoprotein Apo-A2 and CSF-measured human total tau protein was significantly negative (*r* = −0.22, *P* < 0.01). Furthermore, dimethylglycine and citrate were positively correlated (citrate – *r* = 0.25, P_FDR_ < 0.01; DMGly – *r* = 0.25, P_FDR_ < 0.01) with the calculated LEDD dosage.

Overall, there were no clear results from any correlations checked for datasets based on values of MoCA. We can report that the two-group selection of non-PD controls and the PD recessive group provided a positive correlation with 3-hydroxybutyrate, a ketone body metabolite, (*r* = 0.39, P value not significant) and MoCA. Finally, the clinical depression score BDI-II showed a positive correlation with the apolipoprotein ratio ApoB100/ApoA1 (*r* = 0.20, *P* < 0.01; Fig. 4).

Besides the parameters listed in Fig. 3, Nfl plasma and other parameters (Aβ1–42, Aβ1–40, Aβ1–38, P-tau-181, weight, height, all available MoCa values, sex, age, aao – age at onset, PDdd – PD disease duration, CSF NfL levels, MDS UPDRS III score) were checked via correlation analysis as well. However, correlation for those parameters were only marginal (Supplementary Fig. 3, Supplementary Table 3).

In here, we also had to overcome the issue of missing entries with some sub-group merging and summarize the main challenges and how we addressed them. One patient with mitochondrial disease (labeled as ‘Mito’) was diagnosed also PD, so their PDdd value (of PD disease duration) was applied due to their positive PD status. For metadata preparation of tables in correlation analysis, we merged some groups to preserve all available metadata entries and reach the highest n-number. Female controls (control_F) were merged with Mito and PD *LRRK2* groups for alpha-synuclein data. For BMI data, PD *LRRK2* and PD recessive groups were merged. For h-t-tau (h-Tau) data, DLB and PD *LRRK2* groups were merged. For LEDD data, DLB and PD *LRRK2* groups were merged. For MoCA data, DLB and Mito groups were merged. For BDI-II data, non-PD controls were merged with the extra female non-PD controls (Control_F). For Aβ1–40 data, Control_F and DLB groups were merged. For Aβ1–42 and also p-tau-181 data, DLB, Mito, and PD *LRRK2* groups were merged. PD *LRRK2* and PD recessive groups were merged for weight and height characteristics. For PDaao data, the PD *LRRK2* group and the PD-Mito individual were merged. For Aβ1–38 data, Control_F and DLB groups were merged. For Nfl plasma data, Control_F, PD double mutation carrier, and PD *LRRK2* groups were merged. For CSF NfL data, Control_F and Mito groups were merged. For MDS UPDRS-III data, Control_F, DLB, and PD *LRRK2* groups were merged.

To further elaborate how NMR parameters have a potential to distinguish between Sporadic PD early and Sporadic PD late, we performed an area under the curve receiver operating characteristic AUC-ROC analysis on the LEDD dataset exclusively for both early and late PD duration groups. In this aspect, citrate was higher in the late PD group (Fig. 4). However, when checked directly, we could not find citrate as the closest metabolite parameter positively correlated with LEDD. By contrast, blood tyrosine showed a stronger correlation (for early and late PD groups), likely due to their close metabolic positions, as tyrosine is a precursor of L-dopa. Furthermore, for PD genetic groups there was a high positive correlation between LEDD and blood pyruvate. Negative correlations were identified between LEDD in the PD genetic subgroup and several parameters of the HDL4 subfraction.

Regarding the LEDD-driven influence on metabolome, we briefly did a split of LEDD dataset of our study and reclassified with accordance of levels (zero, low, medium, high) and patient sex as possible factors driving our metabolic reported alterations (Supplementary Fig. 4). With an applied threshold of p_FDR_ < 0.001, we saw that most metabolic driver variables were dimethylglycine and citrate. On the lipoprotein part, we can summarize that several groups (re-stratified) were different based on how much of HDL lipoprotein particles, including HDL cholesterol blood values; were there and also the SPC signal.

We furthermore saw abnormal elevation of HDL triglycerides on average in the PD recessive (females) samples. As the following correlation shows, HDLs and also SPC in the strongest way correlate with the female sex factor.

We also did an integrated (correlation) analysis of blood methionine, DMGly and formate levels to check for any abnormalities in one-carbon metabolism in people with PD. We also included some extra parameters and metabolites as of described for the one-carbon metabolism, that we can detect with our methodology, as reviewed in^21^. We also did a correlation analysis to see if there was a connection between DMGly and LEDD in both GBA mutation positive and sporadic PD groups, to work out if DMGly changes can be affected by dopaminergic treatment.

Of note, we were able to see that blood citrate levels follow (by correlation analysis) closer to the factor of PD disease duration – leading metabolite, while patient age factor was positively correlating stronger with other metabolites in places prior to citrate (choline, TMAO, tyrosine, acetate, and dimethylsulfone) and also lipoprotein variable of HDL-1 subfraction signal constitute for apolipoproteins A1 (Supplementary Fig. 3).

Citrate, as any other our NMR parameters, did not meaningfully correlate to the MDS UPDRS-III patient data. Our determined value of correlation coefficient is + 0.013554 (*r*; Spearman’s; Supplementary Table 3).

### Analysis 6: methionine is significantly decreased in subtypes of GBA driven PD

**Fig. 5:**
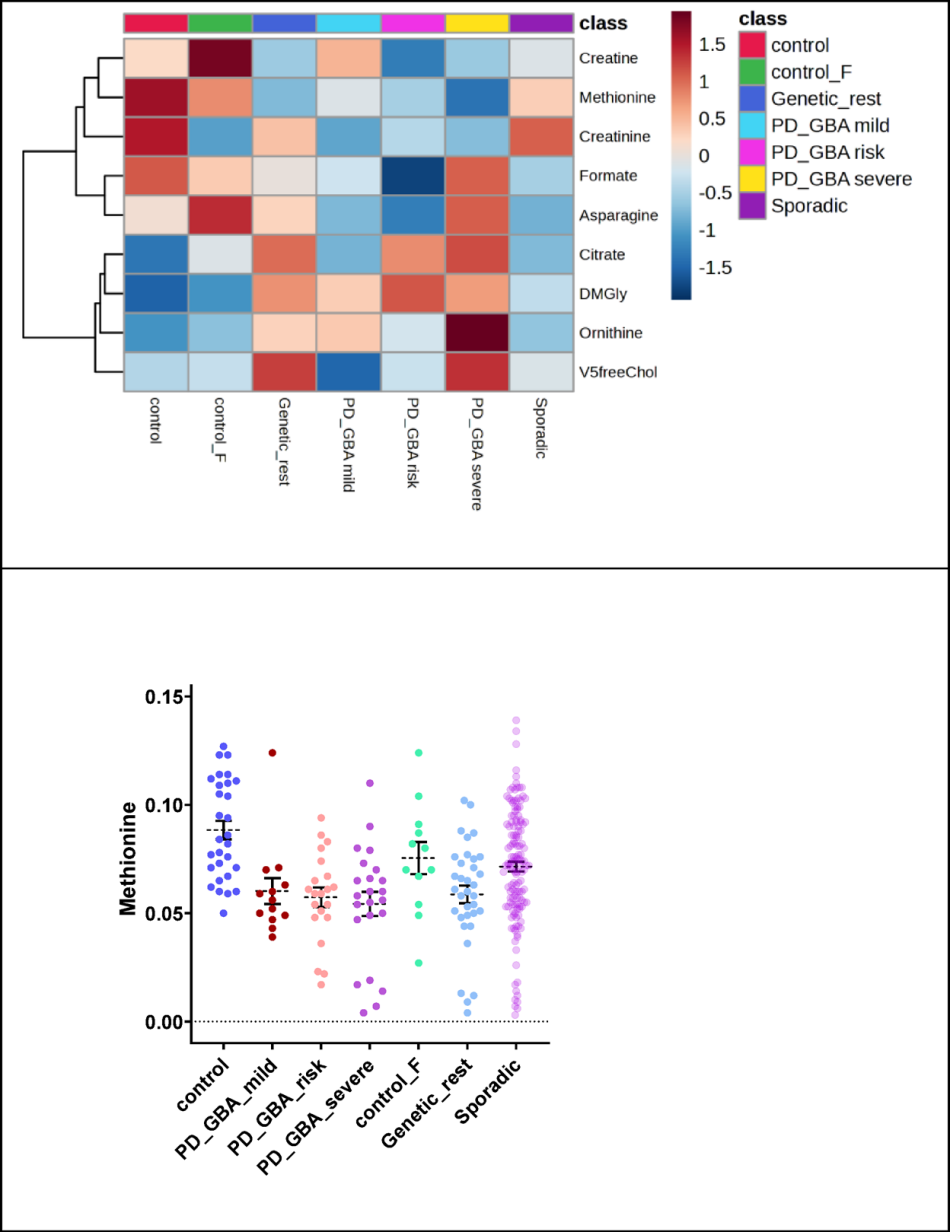
Significant metabolite and lipoprotein alterations in eight-group comparison (non-PD Control, PD genetic phenotype with *GBA* groups excluded, PD sporadic, PD *GBA* groups divided by severity grade). Heatmap of p-value FDR-significant findings checked via ANOVA statistical testing. Dot plot of methionine shows lowest level of this metabolite, shown as means and SEMs, in GBA groups.

Further discrimination within the PD *GBA* group may be of particular clinical interest (Analysis 6 in Fig. 1E), and we identified lowered methionine levels in *GBA* groups (ANOVA, Fig. 5). Unfortunately, the *GBA* VUS group could not be analyzed in Analysis 6 due to the low n-number limitation of the number of analyzed groups. The mentioned group is further checked in Analysis 7.

Metabolites that were distinct in this comparison were creatine, formate, asparagine, citrate, creatinine, ornithine, and DMGly. We also identified the significance of the VLDL-5 subtraction parameter of free cholesterol. It is essential to mention that PD duration covariate (and also sex- and age- based covariates) is helpful for further investigations. Doing so, we found creatine as such dependent metabolite (Supplementary Table 2). The data entries from asparagine have not been analyzed (too many zeros).

### Analysis 7: methionine and ornithine levels differ in diagnosed PD GBA patient groups

Interestingly we also found decreased methionine for *GBA* VUS in addition to the previous analysis. Alterations in methionine can be connected to the gut microbiota^24–26^. Concerning case-control discrimination of *GBA* PD patient samples and risk stratification of the *GBA* mutation (*GBA* risk, mild, severe), methionine was identified as significant over analyzed groups.

**Fig. 6:**
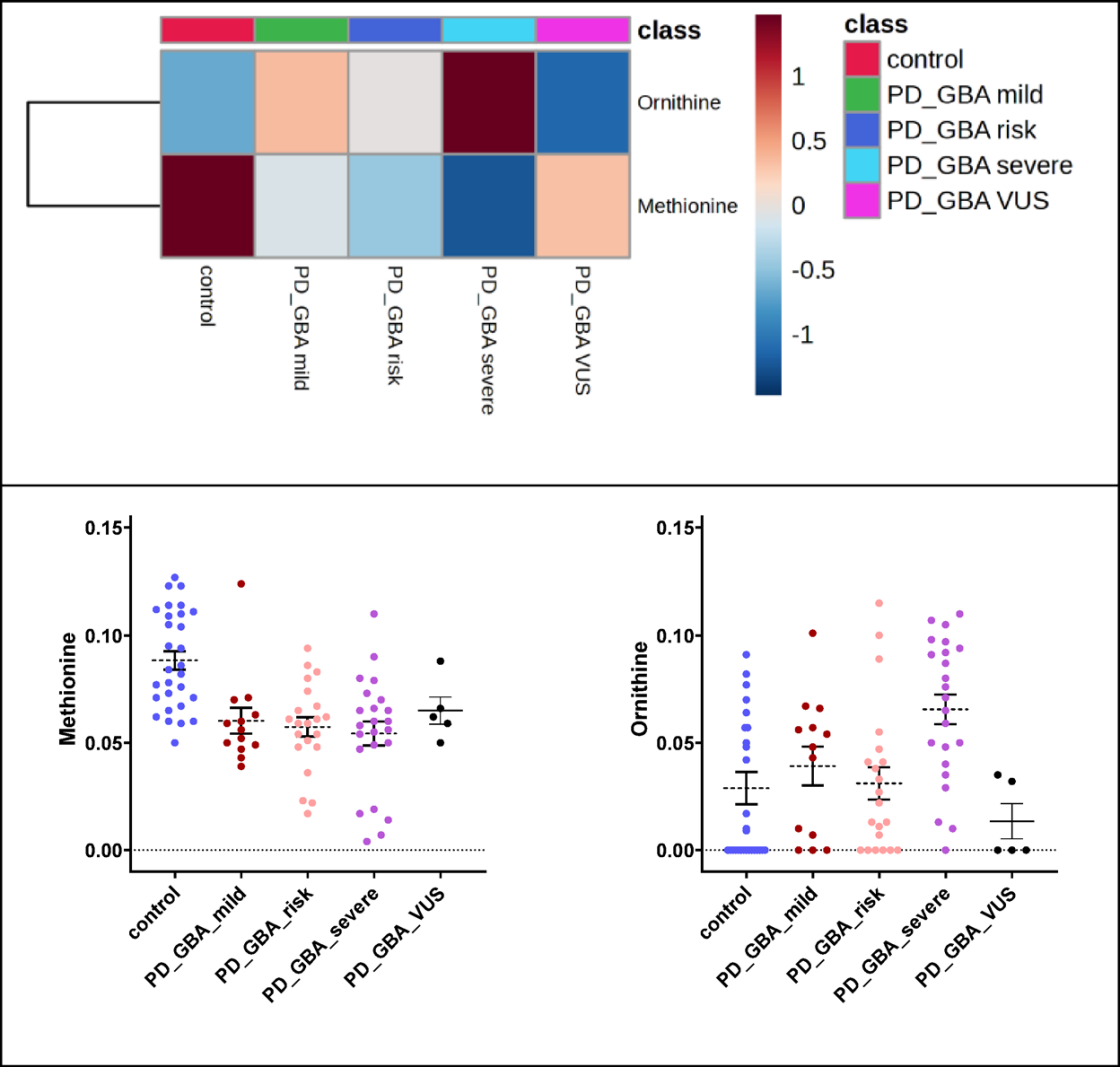
Metabolomic and lipoprotein significant alterations in five-group comparison (non-PD Control, PD *GBA* groups separated by a severity grade). Heatmap of p-value FDR-significant findings checked via ANOVA statistical testing. Cohort groups providing dot plots of ornithine and methionine, shown as means ± SEM.

It was lower in comparison to non-PD controls (Fig. 6). Furthermore, a substancial increase in ornithine levels was shown in *GBA* patient groups. Overall, the PD *GBA* severity grade was significantly (on average) discriminated by ornithine elevation levels. Testing covariance resulted in identification of none of the two metabolites being dependent of PD disease duration time (or age, or sex) rather than discrimination of the analyzed patient groups (Supplementary Table 2).

### Analysis 8: A potential interplay of 2-aminobutyrate in PD recessive patients

We performed additional analyses in Analysis 8 (Fig. 1E) to find potential discriminating metabolites between non-PD controls and PD recessive mutation carriers, including some small patient groups (Table 1). The groups in question here are as follows: PD patient samples with double mutations (carriers), PD *LRRK2* mutation-positive, PD *PRKN* biallelic, PD *PRKN* monoallelic, non-PD controls, non-PD controls the female surplus group, DLB individuals, and the Mito group.

**Fig. 7:**
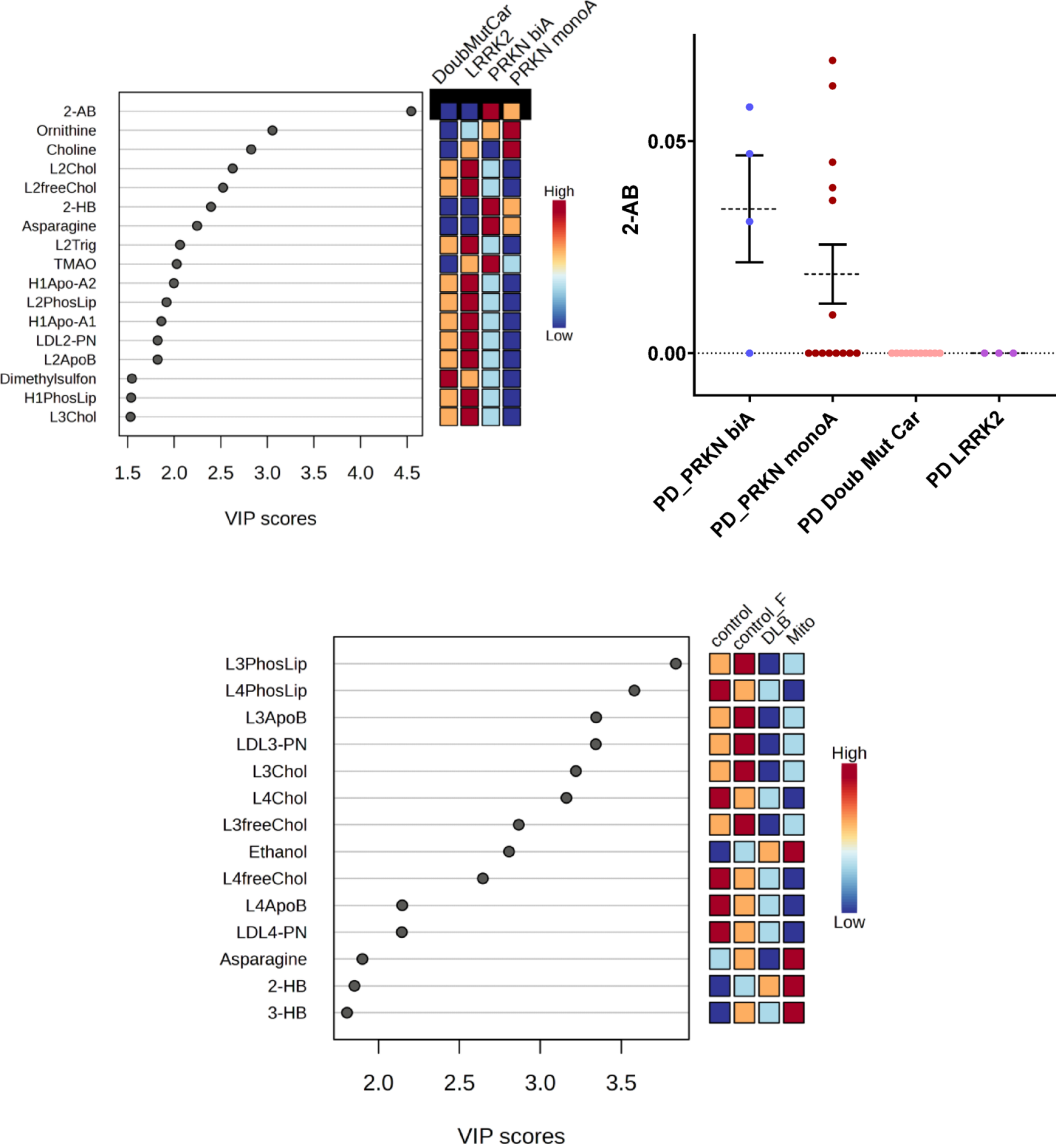
Metabolomic and lipoprotein alterations in the comparison of smaller groups (non-PD Controls, DLB, Mitochondrial diseases, PD double mutation carriers, PD *LRRK2* positive, PD *PRKN* biallelic, and PD *PRKN* monoallelic). Using regression analysis (PLS-DA) 2-aminobutyrate (2-AB, shown as means ± SEM) and, on the other panel, LDL-3 and LDL-4 lipoprotein particles showed strong discriminatory power. PLS-DA – partial least squares discriminant analysis.

In this analysis, we could not find significant differences according to univariate statistics. Thus, to further explore, we applied a regression analysis (PLS-DA) (Fig. 7**)**. In here LDL lipoproteins were shown to be decreased in PD mitochondrial diseases-positive individuals and the DLB phenotype group. Higher 2-aminobutyrate (2-AB) were observed in PD *PRKN*. 2-AB was found at zero levels for the patient groups of PD *LRRK2* positive and the double mutation carrier individuals’ samples (Supplementary Table 1).

## Discussion

In clinical routine, patients and individuals with symptoms of Parkinson’s disease are usually stratified based on genetic testing, imaging, established CSF/blood biomarkers and clinical history. In recent years, scores of clinical questionnaires and tests have been used (e.g., the Unified Parkinson’s Disease Rating Scale (UPDRS) for clinical severity of the main PD symptoms, the Beck Depression Inventory (BDI) for mood assessment and the Parkinson’s Disease Questionnaire (PDQ-39) for subjective disease symptoms)^27–33^. In here, the Montreal Cognitive Assessment (MoCA) and the Mini Mental Status Examination (MMSE) are the tests most widely used for clinical research on PD dementia^34,35^, and could be likely used to diagnose (and improve accuracy in diagnosing) cognitive impairment and dementia in PD.

Of note, it has been shown that various rare mutations in genes causing familial Parkinson’s disease are linked to PD pathogenesis, having led to a broader understanding of PD as a whole, including idiopathic PD (iPD)^36,37^. Thus, to better understand iPD, some studies provide map-like pathological observations of clinical diagnostic data to better understand PD heterogeneity^38–41^. Furthermore, towards the impact of biological sex, the prevalence of PD is higher in males than females^42,43^. This is an additional challenge when aiming for stratification and mapping phenotypic features towards a refined and improved management of PD.

An overall analysis of CSF and blood serum biomarkers in a large (*n* = 453) cohort of PD patients revealed a panel of inflammatory markers and sex-specific differences^44^. Within DJ-1 parkinsonism, an important role is played by altered mitochondrial functions and oxidative stress^45,46^. Furthermore, the *PARK7* mutation is associated with primary parkinsonism and can be disrupted by DJ-1 activity^45,47,48^. Both oxidative stress and energy metabolism are strongly impacted by mitochondria, so the features of these organelles must be considered when assessing imbalanced states in neurodegeneration phenotypes^49–51^. Here, quantitative metabolites levels could help to assess the magnitude of oxidative stress, aging, dietary effects, and overall neurodegeneration.

To evaluate the progress, prognosis and onset of PD, biomarker-based investigations reflecting the intensity of nervous system degeneration are highly relevant. Our present study used a quantitative in vitro diagnostics research (IVDr) NMR spectroscopy approach to measure a targeted panel of blood metabolites and lipoproteins that has to technical potential to be readily applied during routine clinical visits or within extensive population screening program.

Having a set of different PD subgroups, we performed a total of eight different statistical comparisons to assess how NMR parameters can stratify these different patients and to learn what major confounders have to be considered. Patients with recessive PD forms were studied to gain insights into the genetic basis of metabolomic findings derived from blood samples. The analysis focused on mutations associated with the *parkin* (*PRKN*, *PARK2*), *PINK1* (*PARK6*), and *PARK7* (*DJ-1*) genes. The respective proteins from all three are helping to maintain mitochondrial wellbeing and are crucial for the optimal functioning of mitochondria^52–55^.

In the forthcoming discussion sections, we will concentrate on the results that were identified as FDR-rate significant.

### Methionine is among the strongest differentiating metabolites overall

The non-wildtype PD groups (genetic group) demonstrated lowered levels of methionine and histidine. This is in line with another recent PD blood metabolomics study, where the two metabolites were lower on average for advanced stage of PD patient group in contrast to controls^13^. Histidine provides antioxidative protection in the metabolism^56,57^. Methionine is protective against higher levels of ROS^58^ in a model of PD.

Besides methionine, creatinine was another important metabolite in Analysis 1, comparing the five major patient groups of our study. Both compounds were significantly altered in amyotrophic lateral sclerosis (ALS)^59^. The study showed that these two metabolites together with asymmetric dimethylarginine and some phospholipids were machine learning modeled to highlight a trend of metabolically relevant ALS pathogenic alterations in contrast to controls.

### Importance of biomarkers and meta-analysis in PD research

Starting from the methodology of seed amplification assays (SAA), there were indications of alpha-synuclein SAA was checked as the most accurate way to make a diagnosis^60–63^. Xylaki et al. 2023 have reported a significant reviewing work to identify extracellular vesicles (EV) proteins and ribonucleic acids; and EV-derived biomarkers for diagnosing PD and assess their clinical significance^64^. Furthermore, Xie et al. 2023 did a multimodal meta-analysis of the changes of neuroimaging parameters in PD compared with normal controls through whole-brain meta-analysis^65^. There were no signs of significance via multimodal analysis among brain areas of PD patients, as they had series of spontaneous brain activity abnormalities^65^. Yet, a meta-analysis performed by Xu et al. 2023 showed a consistent increase of iron metabolism markers in the brain of PD patients^66^.

### Ornithine alterations might serve as readout for oxidative stress in PD

Evaluating the impact of GBA, methionine and ornithine levels were changed along with the *GBA* mutation risk status. As for ornithine, in PD, it was found to be elevated in urine^67^. A recently published meta-analysis, however, did show PD-related reports on increased ornithine metabolite levels (blood, urine, CSF) compared to controls^68^. Ornithine changes (blood, urine) could indicate increased liver and kidney activity in PD and changes within the urea cycle metabolizing nitrogen.

However, according to one study of L-Ornithine-L-Aspartate^69^, it may prevent neuronal dysfunction in Parkinson’s disease in in-vitro models due to the capability of ornithine to cross the blood-brain barrier. As ornithine is a compound that participates in polyamine metabolism. Polyamines can participate in gene expression and antioxidative processes^70–72^, and ornithine was elevated in serum of PD patients^73,74^. On the other hand, it was proposed that ornithine causes longitudinal osmotic pressure in brain regions and induces damage^74,75^. Elevated ornithine levels were reported in aged (advanced stage) PD patients^76^. Therefore, ornithine could play a significant role in the blood-based screening for PD individuals.

It may also suggest poor digestive function^77,78^ in Parkinson’s disease patients (e.g., resulting in constipation). But, in this case, blood ornithine is reported as lowered in patients with functional constipation^79^. Finally, a urine of idiopathic PD patients’ screening study showed elevated ornithine also in that matrix^67^. Authors of the publication^67^ associated their ornithine finding with patients’ bowel problems, such as constipation, and mitochondrial dysfunction^73,77,80^.

### Significant alterations dimethylglycine could mirror dysbiosis in PD

Several of our comparisons (Analysis 1, Analysis 2, Analysis 6) we found an increase in DMGly. This metabolite is result of choline metabolism and could indicate changes of gut-related metabolism. An increase in DMGly in our study groups could be a distinctive change of gut-related metabolism, which did not follow, however, the previously reported DMGly decrease in PD patients^13^. DMGly contributes to one-carbon metabolism with formic acid and methionine. L-Dopa may be an altering factor in the one-carbon metabolism as well^81^. Regarding one-carbon metabolism, the liver as main entrance organ for most gut-derived metabolites must be considered^82^. Therefore, it would be beneficial to study and compare blood changes and more accurate or frequent data on the metabolites belonging to both the metabolic pathway and metabolic profiling^83^.

### Citrate as a metabolite of aging

Further results Analysis 1 showed elevations of citrate and dimethylglycine in other groups (PD *GBA*, Sporadic PD late, and PD rec) compared to controls and Sporadic PD early participants’ samples. Further studies with larger n-numbers have to support these findings. Overall, higher blood citrate might indicate increased aging^84–86^. There may be a link also towards oxidative stress. Blood citrate was increased in blood of PD, progressive supranuclear palsy, and multiple system atrophy patients when compared to controls^87^.

Changes of citrate refer to TCA cycle metabolism alteration, which (on par with other metabolic pathways) is one of several features in mitochondrial dysfunction and discrimination of Sporadic PD early and Sporadic PD late^88^. Regarding that, our findings of higher citric acid levels in Sporadic PD late are not in line with another NMR-based study of PD patients^89^ and do not meet the direction of changes compared to the de novo drug-naive and advanced stage PD individuals^13^. So, findings upon citrate remaining contradictory.

Our analysis found that there was no significant correlation between the blood citrate levels and the MDS UPDRS-III scores. Citrate and isocitrate did shown a certain tendency to be higher in aged population^90^. This same preprint also found that citrate was higher in Dopa-negative idiopathic PD rather than controls, also there citrate correlated itself in a negative way correlation to the factor of PD severity^90^. Interestingly, one paper suggested to collectively view PD-related evidence of citrate and also tyrosine metabolism alterations^91^ and linking into^92^ towards earlier investigations of PD in cerebrospinal fluid, blood, and saliva^93,94^.

### Biological sex is a major confounder for NMR based serum metabolites and lipoproteins

Studying the impact of biological sex upon NMR-based parameters, VLDL particles were elevated overall in the men of our study, which is a typical trend as before^95–99^. Furthermore, levels of BCAAs were elevated in male subjects^100,101^. This is interesting since we discovered sex-related changes, including creatinine and valine, in our cohorts. Sex-specific differences have been underappreciated, that should no longer happen^102–105^. Multiomics approaches in human tissue research study of ALS and animal model investigations highlighted therapeutically related evidences and sex-related differentiations^106^. We believe that future research of PD neurodegeneration could benefit from further approaches in securing evidences in both therapy- and sex-related alterations.

Sex-specific and PD-related changes in lipoproteins in this study may be explained by starvation rates, insulin resistance, dyslipidemia, and possibly sarcopenia. Sarcopenia has a wide range of associations with patients’ comorbidities, yet specific links have been found how insulin resistance is associated with obesity and sarcopenia^107^.

On another hand of PD study field that is about sex hormones^108^, estrogen use after menopause was associated with a lower risk of developing Parkinson’s disease as reported in^109^. A study^110^ in monkeys showed^111^ that estradiol altered dopamine metabolism^112^ and transporter uptake in the brain after surgically induced menopause.

Measurement of dihydrotestosterone would have allowed better characterization of the metabolism of androgens and reveal more involvements about PD, as was pointed out in^113^. There is some evidence to suggest that drinking alcohol and smoking might reduce the risk of PD for men more than for women^114^. For example, there are differences in how men and women’s bodies react to alcohol and smoking, and also in how much nicotine they can take in^114^. A healthy alternative, physical exercise is a way to reduce PD-associated risks for men and women with also reflections towards their metabolic and lipid alterations^114^.

### NMR parameters show rather week correlations with clinical PD biomarkers

Matching NMR parameters with clinical biomarkers, several correlations provided some interesting findings. Correlation analysis revealed a notable correlation between BMI and the NMR-based Glyc/SPC ratio. This ratio is an NMR-based metric widely during COVID-19 research. Both Glyc and SPC parameters, parts of the ratio, were correlated with the BMI index in a study of COVID-19 patients^115^.

We found that with higher LEDD, slightly higher DMGly levels are observed. This correlation could be linked to gut dysbiosis. Yet, DMGly is also part of the folate cycle metabolism and close to homocysteine via a remethylating pathway^116^. This report rose the idea of high homocysteine in blood may be due to the adverse effects of L-DOPA treatment. The one-carbon metabolism was reported to be connected for PD dementia patients cohort in the direction of higher homocysteine amounts in the brain due to L-DOPA^117^. In some additional clinical context, patients with autism were reported to have a significant increase in DMGly due to oxytocin^118^.

We noted another correlation of LEDD with blood citrate. Moreover, we found blood citrate to well discriminate LEDD-provided datasets of Sporadic PD early and late disease duration groups in the AUC analysis. Overall, other researchers showed via correlation analysis some significant changes in metabolomics that are potentially applicable for further research of idiopathic PD, as for example correlations between UPDRS III motor score and lactate and BCAAs (valine and isoleucine)^123^.

### Evaluation of quantitative IVDr NMR spectroscopy to serve as diagnostic add-on in PD research

One strength of NMR spectroscopy is it is high-throughput and excellent experimental reproducibility when investigating large cohorts of human samples allowing to combine data from different batches. Furthermore, the absolute quantitation allows a direction comparison with clinical metadata and lab parameters or protein-based biomarkers. Blood research, in this approach, may provide further insights of PD pathology^119^. Furthermore, the combination of NMR-based data sets of multiple biofluids from PD patients with knowledge of the underlying genetic PD predispositions (including familial dominant and recessive forms of PD), may provide further context to previous findings in PD patients with *PINK1* mutations^120^.

A recent NMR-based PD blood metabolomics study reported elevated plasma pyruvate levels^121^. Additional results from the same study showed a significant decrease in TCA cycle metabolites, suggesting the importance of underlying mitochondrial dysfunction. However, a limitation of that study was the relatively small number of participants. Another study identified elevated plasma levels of the branched-chain amino acids (BCAAs) valine and isoleucine^87^. BCAAs may reflect pathophysiological changes in the blood of individuals with neurodegenerative diseases that have been reported as well for patients with dementia^16,122^. Another metabolite that was elevated in PD blood was glutamate^87^ which is part of the alanine-aspartate-glutamate metabolism pathway. Comparing iPD patients with healthy controls^123^, higher serum levels of alanine, citrate, acetate, glycerophosphocholine, glycine, and trimethylamine-N-oxide (TMAO) were found in iPD. Interestingly, negative correlations were reported between BCAAs (and lactate) and UPDRS III motor score next to another negative correlation between TMAO and disease duration^123^. Alanine is involved in muscle metabolism. Which is interesting, as loss of muscle tissue (sarcopenia) is an aging parameter also in Parkinson’s disease^124–126^.

Amino acids transported to the brain (via the blood-brain barrier) are necessary to sustain essential brain functions and BCAAs contribute to a vital part of our brain energy metabolism^87^. In a study from 2020, patients with PD were investigated by NMR metabolomics^127^. It was demonstrated that acetate, ketone bodies (acetone and 3-hydroxybutyrate (3-HB)), lysine, glutamine, tyrosine, and phenylalanine were increased in PD with a decrease of glutamate^127^. In a later study of 2022, an NMR standard operation procedure (SOP^128–130^ solution was used to profile metabolites and lipoproteins in blood samples of de novo drug-naive PD patients, healthy controls, and advanced PD patients with dopaminergic treatment^13^. However, the evidence of PD progressive patients’ alternated blood phenotype was drawn to oxidative stress factors in Meoni et al. 2022 ^42^. Later, the study researchers created a biological age prediction model in PD patients to investigate the discrepancies observed between age estimation in control individuals and age prediction in patients via NMR-based metabolomics^14^.

In recent years, metabolomics and lipidomics have emerged as promising analytical tools to measure human biofluids upon health and disease status. IVDr NMR spectroscopy is a method of choice as it is highly reproducible, quantitative, and high throughput. IVDr NMR-based studies have been successfully applied, especially for metabolic disorders such as diabetes, cardiovascular disease^131^, or hypertension^132^. Under the hypothesis that the duration and severity of PD are impacted also by metabolism, we gathered 287 blood serum samples within three different subprojects.

We merged these cohorts for a total of 8 different analysis. Though we estimated that statistical power would be sufficient, only a handful of metabolites and lipoproteins met significance threshold for multiple hypothesis testing correction (FDR adjustment). Still, our results demonstrate a first step how IVDr NMR could contribute to future PD screening, progress monitoring or treatment efficacy testing programs. Future investigations should include higher n-numbers for the respective subgroups as within many of our statistical tests we found clear trends of changing metabolites, however p-values after FDR correction remained > 0.05. This is mainly for blood serum metabolite and lipoprotein analysis, which is crucial as these parameters can quickly change to diet and exercise, and a perfect standardized collection is almost impossible. In conclusion, we could give evidence for several significantly changed metabolites and lipoproteins in different PD patient groups, which shows that the impact of metabolism should not be neglected in PD research and that to do so, IVDr NMR serum spectroscopy is a suited tool.

### Limitations of the study

Study design: This project was designed as a metabolomics study. It lacks a holistic clinical metadata knowledge, such as imaging experiments or further clinical laboratory results, which may either not be available or not yet completed, or which otherwise can only be analyzed in a different - time-dependent - type of patients’ analysis. We were also not able to locate all the clinical data from the measured samples as a form of complete matrix. Therefore, several important PD and dementia parameters were represented with a much lower n-number due to the aforementioned data unavailability.

NMR technology: Despite all the recent advantages of NMR spectroscopy, it is well known that the use of complex mixtures such as biofluids comes at the cost of low sensitivity of the method, despite its accuracy. Indeed, the range of reference proton resonance signal concentrations is in the millimolar range. Additionally, overlapping signals during an analysis comes at the cost of some larger error discrepancies.

Sample collection: Next, the use of patient samples for metabolomics studies always is threatened by delayed collection and deep-freezing which could result in the degradation of certain metabolites. Unfortunately, there is no easy way to quickly take a sample of cerebrospinal fluid or any other fluid close to the human brain. Additionally, when a blood or CSF sample is taken, disinfectant alcohols may be introduced into the sample.

Confounder analysis: Age matching between groups was not fully achieved. This is due to the specific nature of the study involving an older population with PD (PD_late). Efforts were made to minimize sudden age differences and to extract statistical data analysis that was fully relevant to the present study. Yet, to make sure our findings are reflective of the disease progression, we ran generalized linear model (GLM) in R (version 4.2.1) and R-studio (2022.2.3.492) with covariates, such as sex, age, and PD disease duration (PDdd).

## Methods

### Cohort description

The present investigation was performed in accordance with the Declaration of Helsinki. All participants gave their written informed consent. Samples for the current NMR study were obtained from the Neuro-Biobank Tuebingen and the DZNE Bonn. The code reference of the study is TUEPAC^133^, Tübingen data, and Bonn reference codes are DESCRIBE and DANCER^134^. CSF amyloid and tau data are also referenced in^135,136^.

Patients were classified, in our present study, as having sporadic PD early if PD endured within five years, or sporadic PD late if endured for more than five years.

Clinical parameters were received in form of an incomplete matrix (datasets with missing measurements) and considered for separate analysis: age at onset and disease duration (analyzed as full years, Supplementary Table 1, Table 1), weight (total *n* = 174 complete records, Cohorts 2 & 3), height and body mass index (BMI, total *n* = 173 complete records, Cohorts 2 & 3), Montreal Cognitive Assessment Index Score^137^ (MoCA, total *n* = 240, Cohorts 1, 2, & 3), MDS-Unified Parkinson’s Disease Rating Scale^33^ section III (UPDRS-III, total *n* = 221, non-MDS ratings^138^ (*n* = 14) were not used, Cohorts 1, 2, & 3), Beck Depression Inventory–II^139^ (BDI-II, total *n* = 172, Cohorts 1, 2, & 3), L-Dopa equivalent daily dose (LEDD, total *n* = 259, Cohorts 1, 2, & 3), CSF-measured neurofilament light chain protein level (Nfl, total *n* = 111, Cohorts 1, 2, & 3), plasma-measured Nfl (total *n* = 38, Cohort 2), CSF-measured α-synuclein (total *n* = 77, Cohorts 1 & 3), CSF-measured Aβ38 (total *n* = 35, Cohort 2), CSF-measured Aβ40 (total *n* = 36, Cohorts 2 & 3), CSF-measured Aβ42 and human total tau (total *n* = 151, Cohorts 1, 2, & 3), p-tau-181 (total *n* = 150, Cohorts 1, 2, & 3). In other words, with each parameter measured for a certain n number of individuals’ samples statistical analysis was measured based on the finalized table with the n number of parameter entries and measured NMR data.

Additionally, biological replicate samples (for the same patient at another timepoint, *n* = 16 patients, 32 samples, Subproject Cohort 3, Supplementary Table 1) were taken and cross-cohort biological replicates from Cohort 2 (marked with #, *n* = 5 patients, 10 samples) as well as from Subproject Cohort 1 (marked with @, *n* = 2 patients, 5 samples). Therefore, the part of samples corresponding to a same individual but at different time point taken were used in the present study.

To exclude sex as a confounding factor, surplus female controls were randomly selected to be excluded from the Controls group. The resulting female control were labeled as “Control_F” group (Fig. 1).

### Quantitative NMR-based metabolite and lipoprotein analysis

Blood aliquots were stored at − 80 °C and transported on dry ice until preparation for NMR analysis. All samples were prepared according to a commercial SOP for blood plasma and serum (AVANCE IVDr Methods, Bruker BioSpin GmbH & Co. KG, Ettlingen, Germany). On the day of preparation, the samples were thawed to room temperature and prepared. The annotation and quantification of serum spectra are based on server-based service from Bruker BioSpin GmbH & Co. KG (Ettlingen, Germany). Nuclear magnetic resonance experiments were accomplished on a Bruker Avance III HD 600 MHz NMR spectrometer (Bruker BioSpin AG, Fällanden, Switzerland). Samples were measured with a 5 mm TXI probe, using Bruker TopSpin, including additionally required IVDr SOP experiments and software plug-ins as provided by Bruker BioSpin GmbH & Co. KG, Ettlingen, Germany.

The data obtained contained a total number of 39 compounds (incl., ethanol and glycerol) and metabolites (via Bruker IVDr Quantification In Plasma/Serum, B.I.Quant-PS™, analysis package), and a total of 112 lipoprotein parameters (via Bruker IVDr Lipoprotein Subclass Analysis B.I. LISA™ (Bruker BioSpin GmbH & Co. KG, Ettlingen, Germany). We also had utilized data on glycoprotein signals from NMR and also supramolecular phospholipid composite signal (SPC) as provided by a Bruker service called PhenoRisk PACS™ RuO (intented for research use only; Bruker BioSpin GmbH & Co. KG, Ettlingen, Germany).

More information on the used Bruker solutions, NMR metabolomics and some applications in research was recently published^140^.

### Statistical analysis

Data was analyzed with the MetaboAnalyst 6.0 (online-based) package for statistical analysis and meta-analysis^22^. Logarithmic scaling was applied. GraphPad Prism software version 10.1.1 (323) was used for plot visualization and age-based comparisons after age-matching of the merged clinical cohorts (see also Supplementary Fig. 5). For each comparison, the following set of parameters was determined: p-values (student’s non-parametric t-testing (via Wilcoxon rank-sum testing method) and non-parametric ANOVA (via Kruskal Wallis Testing method) analysis of variance), and Spearman correlation coefficients (used in PatternHunter). For the t testing, all of the described results were significant via unequal n-numbers check. Graphs (mean ± SEM format) were produced based on input values and generated in GraphPad Prism 10.1.1 (323) for displaying metabolomics data.

Additionally, covariance analysis was done with replacing zeros as missing values and subsequent imputation. We used a generalized linear model (GLM) in R (version 4.2.1) and R-studio (2022.2.3.492) in order to determine whether the covariates, such as sex, age, and PDdd (PD disease duration) acted as confounders. We used GLM with Gaussian link and gamma log link function for normally distributed and skewed data, respectively. Of note, covariate adjusted p-value less than 0.05 is considered significant and such covariates did not confound the association between the group and dependent variable. The test recalculated zeros as missing values and no logarithmic scaling. The primary study statistics are not.

A regression model (PLS-DA, partial least squares discriminant analysis) was used, in short, to establish ranking (via VIP values, variable importance in projection) as discriminatory features of the first component of the regression model score performed for two or more patient groups. The operated threshold for VIP values was > 1.50, and these values were considerably important for reporting.

Several figures in the current work were created with BioRender.com online-based service.

## Electronic supplementary material

Below is the link to the electronic supplementary material.

Supplementary Material 1

Supplementary Material 2

Supplementary Material 3

Supplementary Material 4

## Data Availability

Raw NMR quantitation data (metabolites, lipoproteins and inflammation markers) and basic clinical metadata is included in Sup. Table 1. Further clinical data beyond the information in the manuscript and supplement files is available upon request to: thomas.gasser@uni-tuebingen.de.

## Declarations

### Metadata declaration

The data were provided by the study group of DESCRIBE and DANCER studies of the clinical research of the German Center for Neurodegenerative Diseases (DZNE). And data were provided by the study groups of TUEPAC study at the University Hospital of Tuebingen. The clinical data supporting the findings are available upon request to: thomas.gasser@uni-tuebingen.de.
