# Supplementary material for: Application of IVDr NMR spectroscopy to stratify Parkinson’s disease with absolute quantitation of blood serum metabolites and lipoproteins
Source: Sci Rep. 2025 May 22;15:17738. doi: 10.1038/s41598-025-01352-0 (PMC12098827; doi:10.1038/s41598-025-01352-0)
Supplement: Supplementary file 1 — Supplementary Material 1 [file 41598_2025_1352_MOESM1_ESM.pdf]

Supplementary Material

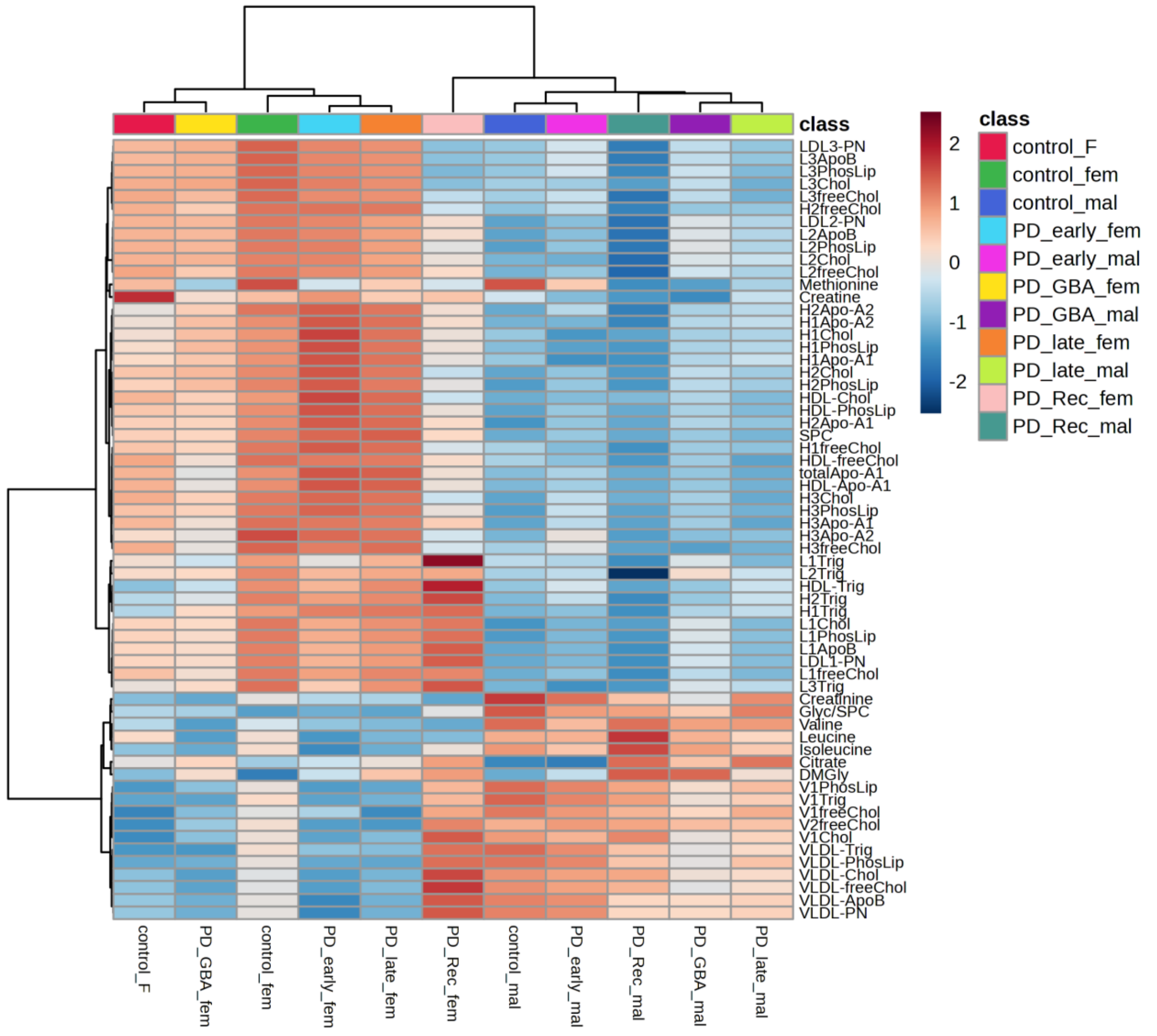

Supplementary Figure 1: Metabolomic and lipoprotein significant alterations in the whole cohort of participants' samples (averaged) analyzed. Heatmap of FDR-significant findings checked via ANOVA statistical testing ($p < 0.001$ shown) divided into female and male sub-cohorts.

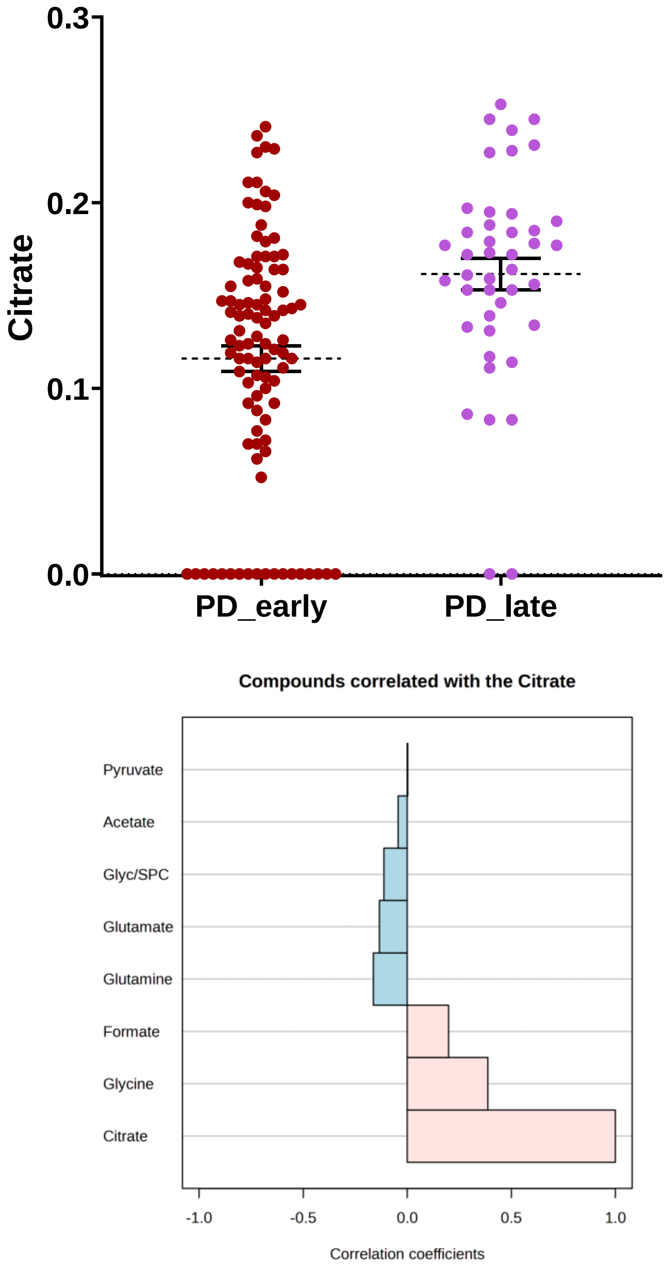

Supplementary Figure 2: Two-group comparison of the Sporadic PD early and late patient samples via t-testing (FDR-adjusted; mean ± SEM format) demonstrating elevation of citrate in the Sporadic PD late group. Pattern-wise correlation plot (Spearman's *r*) was also visualized to represent that our NMR-based citric acid recording correlated rather strongly to other metabolites, in boundaries of the KEGG's defined set of metabolites as Glyoxylate and dicarboxylate metabolism that includes citrate. Glyc/SPC represents a glycoprotein NMR-measured value of relevance to inflammation indication in patient blood.

A

B

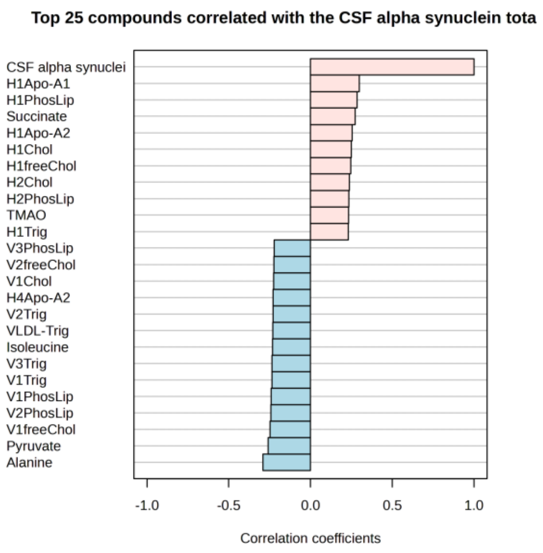

Top 25 compounds correlated with the CSF alpha synuclein tota
CSF alpha synuclei
H1Apo-A1
H1PhosLip
Succinate
H1Apo-A2
H1Chol
H1freeChol
H2Chol
H2PhosLip
TMAO
H1Trig
V3PhosLip
V2freeChol
V1Chol
H4Apo-A2
V2Trig
VLDL-Trig
Isoleucine
V3Trig
V1Trig
V1PhosLip
V2PhosLip
V1freeChol
Pyruvate
Alanine
-1.0
-0.5
0.0
0.5
1.0
Correlation coefficients

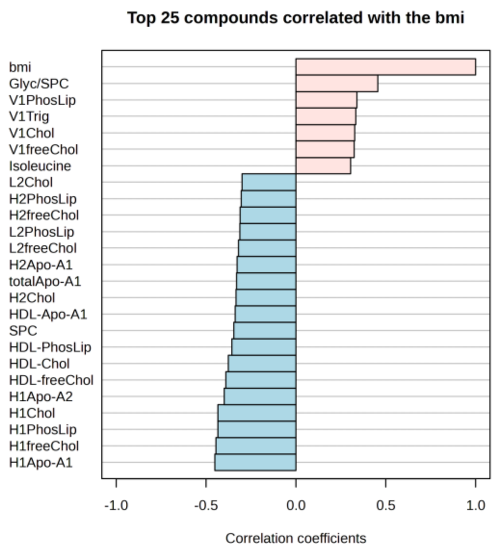

Top 25 compounds correlated with the bmi
bmi
Glyc/SPC
V1PhosLip
V1Trig
V1Chol
V1freeChol
Isoleucine
L2Chol
H2PhosLip
H2freeChol
L2PhosLip
L2freeChol
H2Apo-A1
totalApo-A1
H2Chol
HDL-Apo-A1
SPC
HDL-PhosLip
HDL-Chol
HDL-freeChol
H1Apo-A2
H1Chol
H1PhosLip
H1freeChol
H1Apo-A1
-1.0
-0.5
0.0
0.5
1.0
Correlation coefficients

C

D

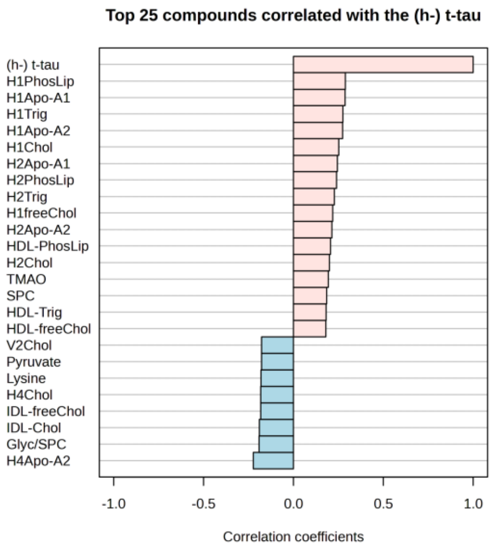

Top 25 compounds correlated with the (h-) t-tau
(h-) t-tau
H1PhosLip
H1Apo-A1
H1Trig
H1Apo-A2
H1Chol
H2Apo-A1
H2PhosLip
H2Trig
H1freeChol
H2Apo-A2
HDL-PhosLip
H2Chol
TMAO
SPC
HDL-Trig
HDL-freeChol
V2Chol
Pyruvate
Lysine
H4Chol
IDL-freeChol
IDL-Chol
Glyc/SPC
H4Apo-A2
-1.0
-0.5
0.0
0.5
1.0
Correlation coefficients

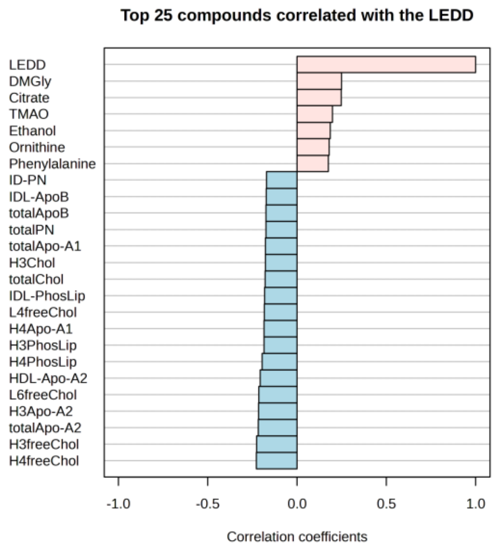

Top 25 compounds correlated with the LEDD
LEDD
DMGly
Citrate
TMAO
Ethanol
Ornithine
Phenylalanine
ID-PN
IDL-ApoB
totalApoB
totalPN
totalApo-A1
H3Chol
totalChol
IDL-PhosLip
L4freeChol
H4Apo-A1
H3PhosLip
H4PhosLip
HDL-Apo-A2
L6freeChol
H3Apo-A2
totalApo-A2
H3freeChol
H4freeChol
-1.0
-0.5
0.0
0.5
1.0
Correlation coefficients

E

F

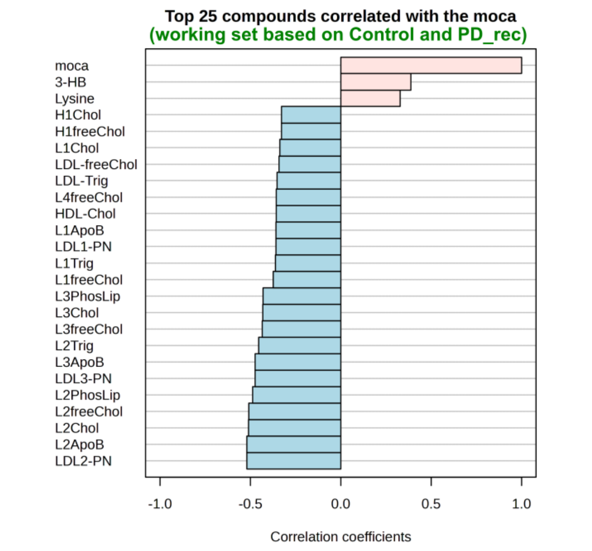
Top 25 compounds correlated with the moca
(working set based on Control and PD_rec)
moca
3-HB
Lysine
H1Chol
H1freeChol
L1Chol
LDL-freeChol
LDL-Trig
L4freeChol
HDL-Chol
L1ApoB
LDL1-PN
L1Trig
L1freeChol
L3PhosLip
L3Chol
L3freeChol
L2Trig
L3ApoB
LDL3-PN
L2PhosLip
L2freeChol
L2Chol
L2ApoB
LDL2-PN
-1.0
-0.5
0.0
0.5
1.0
Correlation coefficients

G

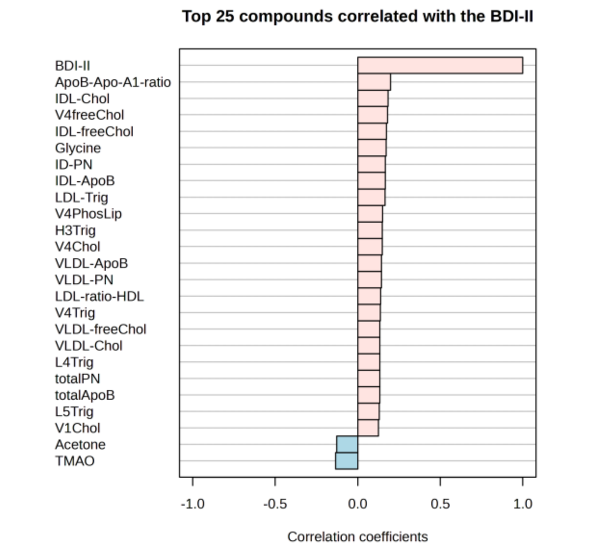
Top 25 compounds correlated with the BDI-II
BDI-II
ApoB-Apo-A1-ratio
IDL-Chol
V4freeChol
IDL-freeChol
Glycine
ID-PN
IDL-ApoB
LDL-Trig
V4PhosLip
H3Trig
V4Chol
VLDL-ApoB
VLDL-PN
LDL-ratio-HDL
V4Trig
VLDL-freeChol
VLDL-Chol
L4Trig
totalPN
totalApoB
L5Trig
V1Chol
Acetone
TMAO
-1.0
-0.5
0.0
0.5
1.0
Correlation coefficients

H

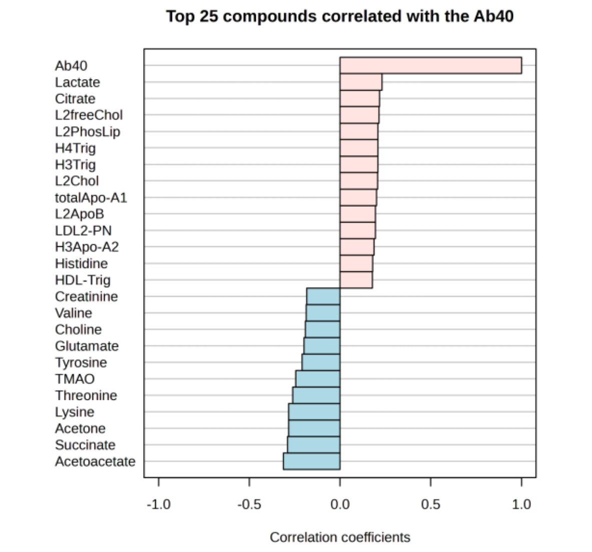
Top 25 compounds correlated with the Ab40
Ab40
Lactate
Citrate
L2freeChol
L2PhosLip
H4Trig
H3Trig
L2Chol
totalApo-A1
L2ApoB
LDL2-PN
H3Apo-A2
Histidine
HDL-Trig
Creatinine
Valine
Choline
Glutamate
Tyrosine
TMAO
Threonine
Lysine
Acetone
Succinate
Acetoacetate
-1.0
-0.5
0.0
0.5
1.0
Correlation coefficients

I

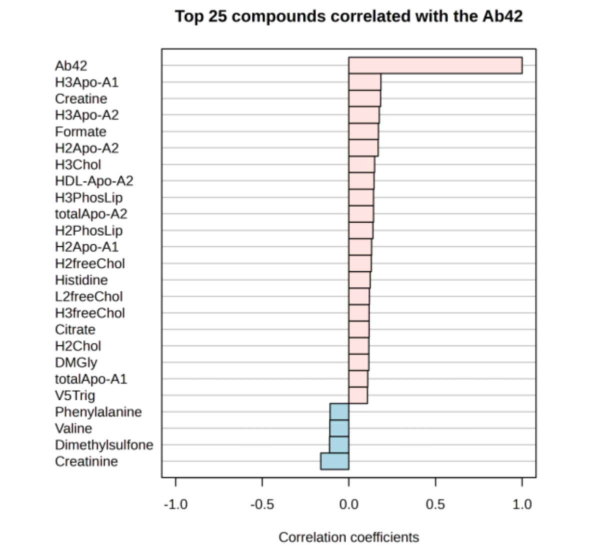
Top 25 compounds correlated with the Ab42
Ab42
H3Apo-A1
Creatine
H3Apo-A2
Formate
H2Apo-A2
H3Chol
HDL-Apo-A2
H3PhosLip
totalApo-A2
H2PhosLip
H2Apo-A1
H2freeChol
Histidine
L2freeChol
H3freeChol
Citrate
H2Chol
DMGly
totalApo-A1
V5Trig
Phenylalanine
Valine
Dimethylsulfone
Creatinine
-1.0
-0.5
0.0
0.5
1.0
Correlation coefficients

J

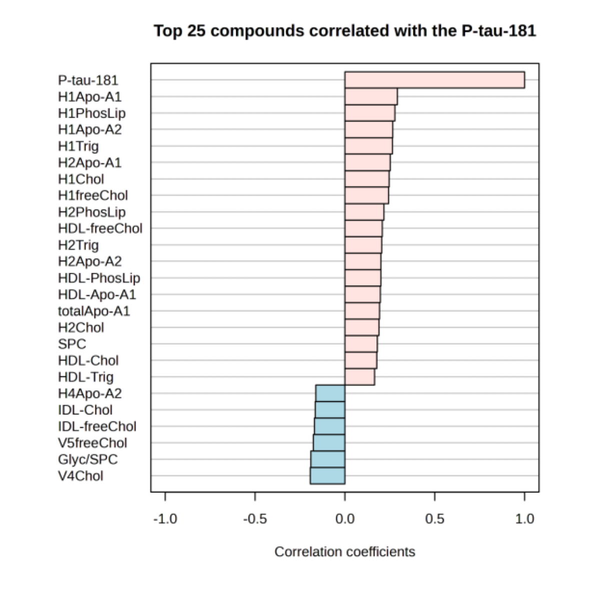
Top 25 compounds correlated with the P-tau-181
P-tau-181
H1Apo-A1
H1PhosLip
H1Apo-A2
H1Trig
H2Apo-A1
H1Chol
H1freeChol
H2PhosLip
HDL-freeChol
H2Trig
H2Apo-A2
HDL-PhosLip
HDL-Apo-A1
totalApo-A1
H2Chol
SPC
HDL-Chol
HDL-Trig
H4Apo-A2
IDL-Chol
IDL-freeChol
V5freeChol
Glyc/SPC
V4Chol
-1.0
-0.5
0.0
0.5
1.0
Correlation coefficients

K

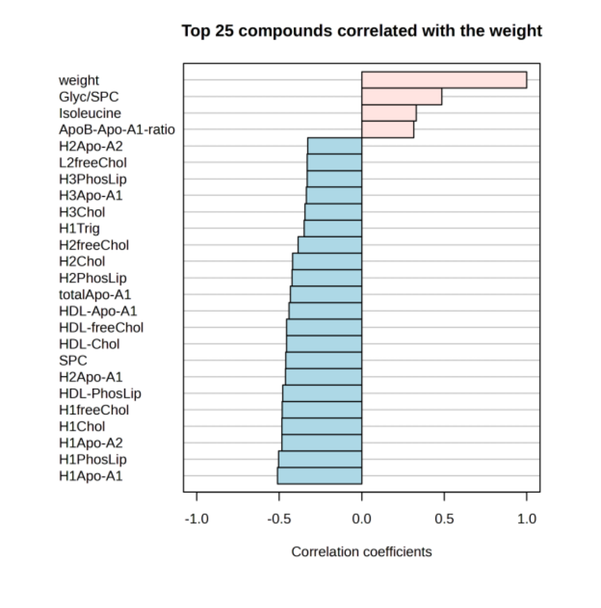
Top 25 compounds correlated with the weight
weight
Glyc/SPC
Isoleucine
ApoB-Apo-A1-ratio
H2Apo-A2
L2freeChol
H3PhosLip
H3Apo-A1
H3Chol
H1Trig
H2freeChol
H2Chol
H2PhosLip
totalApo-A1
HDL-Apo-A1
HDL-freeChol
HDL-Chol
SPC
H2Apo-A1
HDL-PhosLip
H1freeChol
H1Chol
H1Apo-A2
H1PhosLip
H1Apo-A1
-1.0
-0.5
0.0
0.5
1.0
Correlation coefficients

L

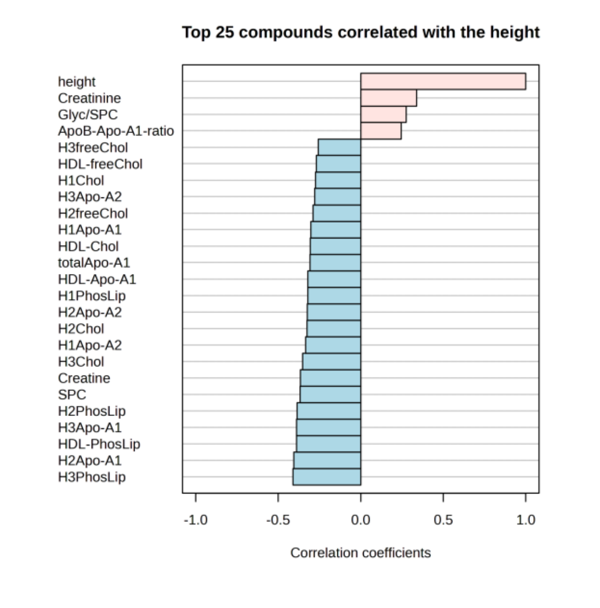
Top 25 compounds correlated with the height
height
Creatinine
Glyc/SPC
ApoB-Apo-A1-ratio
H3freeChol
HDL-freeChol
H1Chol
H3Apo-A2
H2freeChol
H1Apo-A1
HDL-Chol
totalApo-A1
HDL-Apo-A1
H1PhosLip
H2Apo-A2
H2Chol
H1Apo-A2
H3Chol
Creatine
SPC
H2PhosLip
H3Apo-A1
HDL-PhosLip
H2Apo-A1
H3PhosLip
-1.0
-0.5
0.0
0.5
1.0
Correlation coefficients

M

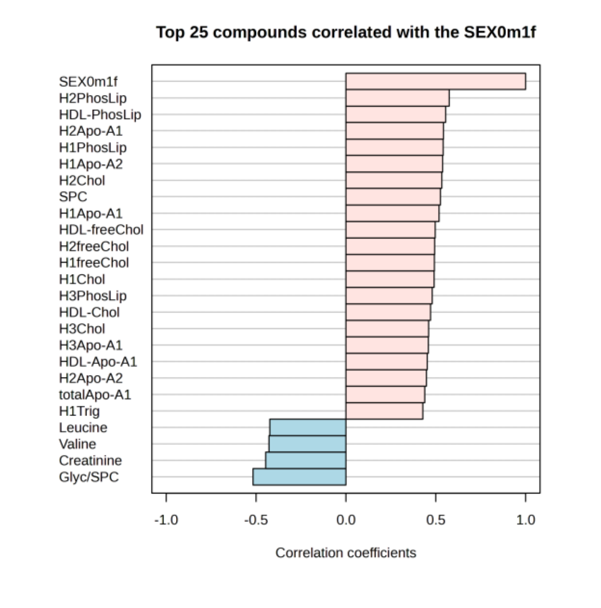
Top 25 compounds correlated with the SEX0m1f
SEX0m1f
H2PhosLip
HDL-PhosLip
H2Apo-A1
H1PhosLip
H1Apo-A2
H2Chol
SPC
H1Apo-A1
HDL-freeChol
H2freeChol
H1freeChol
H1Chol
H3PhosLip
HDL-Chol
H3Chol
H3Apo-A1
HDL-Apo-A1
H2Apo-A2
totalApo-A1
H1Trig
Leucine
Valine
Creatinine
Glyc/SPC
-1.0
-0.5
0.0
0.5
1.0
Correlation coefficients

N

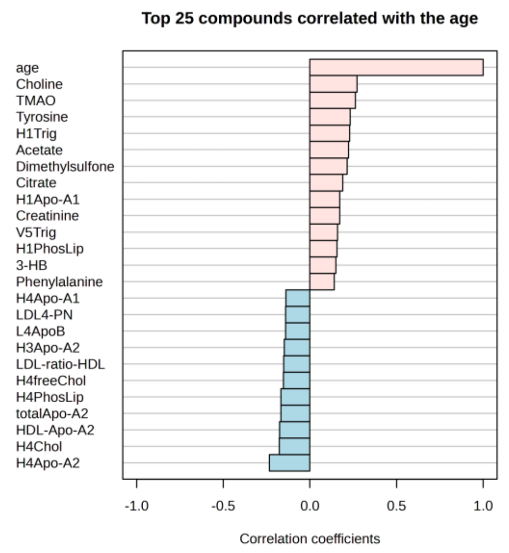
Top 25 compounds correlated with the age
age
Choline
TMAO
Tyrosine
H1Trig
Acetate
Dimethylsulfone
Citrate
H1Apo-A1
Creatinine
V5Trig
H1PhosLip
3-HB
Phenylalanine
H4Apo-A1
LDL4-PN
L4ApoB
H3Apo-A2
LDL-ratio-HDL
H4freeChol
H4PhosLip
totalApo-A2
HDL-Apo-A2
H4Chol
H4Apo-A2
-1.0
-0.5
0.0
0.5
1.0
Correlation coefficients

O

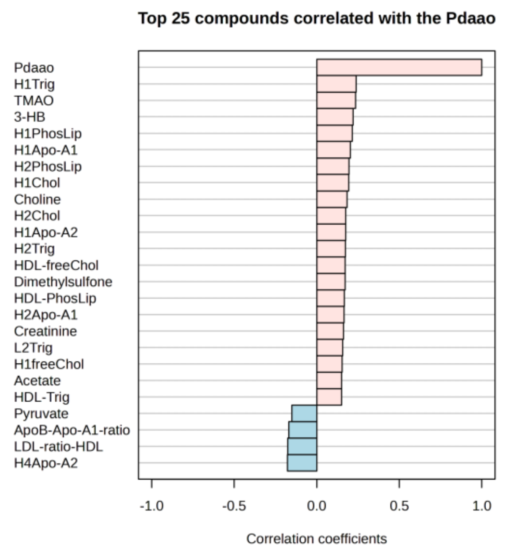
Top 25 compounds correlated with the Pdaao
Pdaao
H1Trig
TMAO
3-HB
H1PhosLip
H1Apo-A1
H2PhosLip
H1Chol
Choline
H2Chol
H1Apo-A2
H2Trig
HDL-freeChol
Dimethylsulfone
HDL-PhosLip
H2Apo-A1
Creatinine
L2Trig
H1freeChol
Acetate
HDL-Trig
Pyruvate
ApoB-Apo-A1-ratio
LDL-ratio-HDL
H4Apo-A2
-1.0
-0.5
0.0
0.5
1.0
Correlation coefficients

P

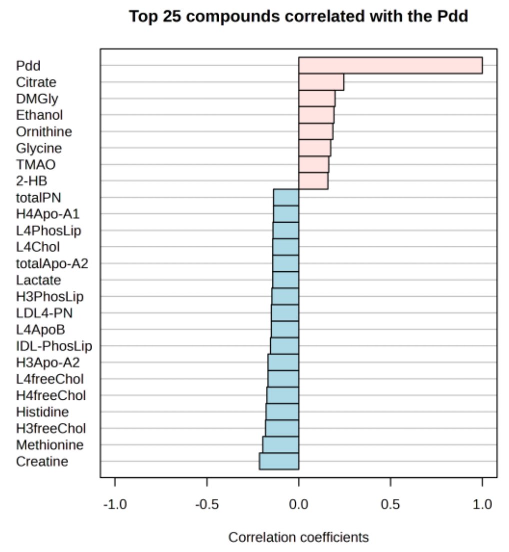
Top 25 compounds correlated with the Pdd
Pdd
Citrate
DMGly
Ethanol
Ornithine
Glycine
TMAO
2-HB
totalPN
H4Apo-A1
L4PhosLip
L4Chol
totalApo-A2
Lactate
H3PhosLip
LDL4-PN
L4ApoB
IDL-PhosLip
H3Apo-A2
L4freeChol
H4freeChol
Histidine
H3freeChol
Methionine
Creatine
-1.0
-0.5
0.0
0.5
1.0
Correlation coefficients

Q

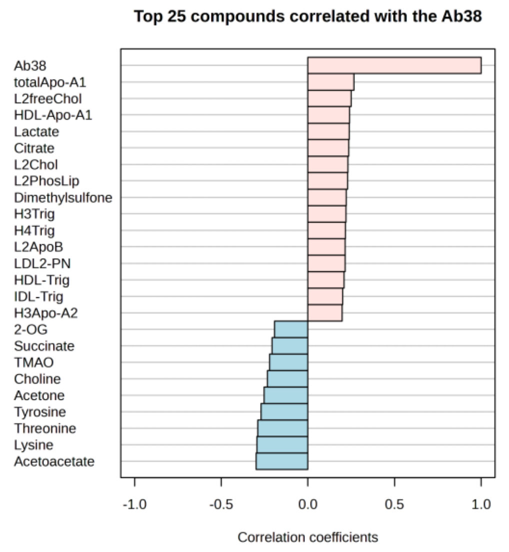
Top 25 compounds correlated with the Ab38
Ab38
totalApo-A1
L2freeChol
HDL-Apo-A1
Lactate
Citrate
L2Chol
L2PhosLip
Dimethylsulfone
H3Trig
H4Trig
L2ApoB
LDL2-PN
HDL-Trig
IDL-Trig
H3Apo-A2
2-OG
Succinate
TMAO
Choline
Acetone
Tyrosine
Threonine
Lysine
Acetoacetate
-1.0
-0.5
0.0
0.5
1.0
Correlation coefficients

R

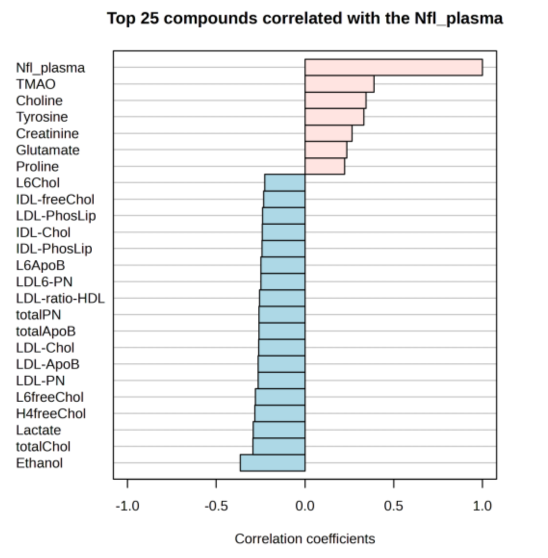

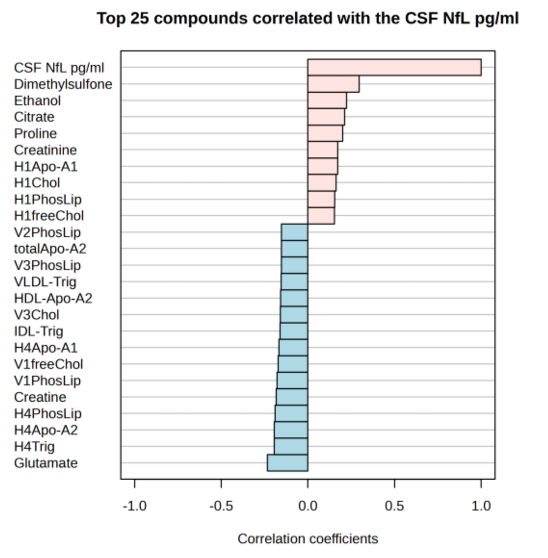

S

T

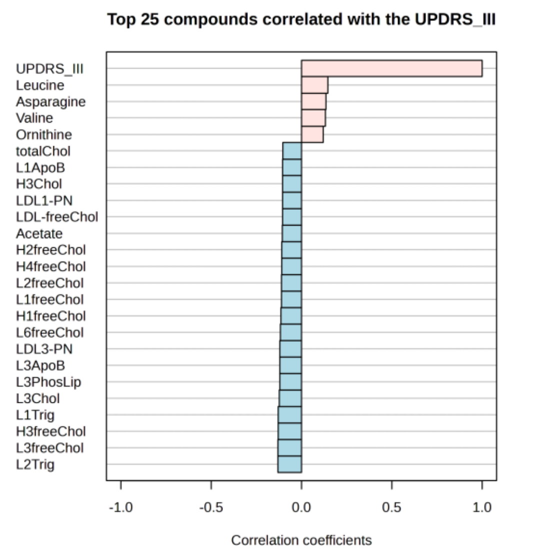

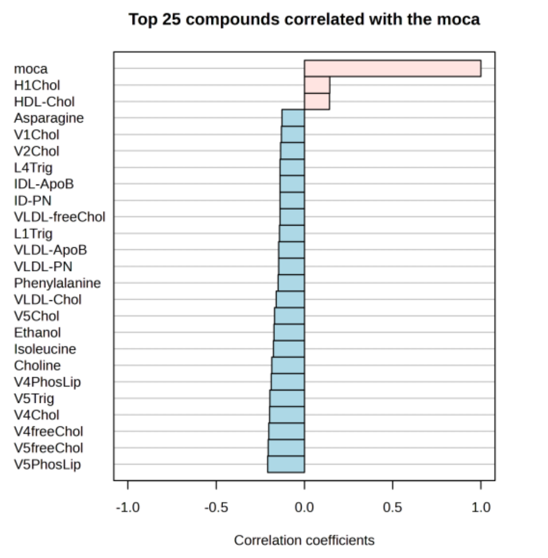

Supplementary Figure 3: NMR-measured metabolites and lipoprotein parameters correlated with different factors. Spearman's correlations are plotted, the inquired parameters are also shown at the top of each graphic with the projected correlation coefficient of r = 1.00. (**a**) CSF alpha-synuclein, (**b**) BMI index, (**c**) human (h-) total (t-) tau, (**d**) LEDD, (**e**) MoCA (Control and PD recessive groups only), (**f**) BDI-II, (**g**) Aβ1-40, (**h**) Aβ1-42, (**i**) P-tau-181, (**j**) weight, (**k**) height, (**l**) sex (binary), (**m**) age, (**n**) aao (age on onset of PD), (**o**) PDdd (PD disease duration), (**p**) Aβ1-38, (**q**) Nfl plasma, (**r**) CSF NfL, (**s**) MDS UPDRS-III, (**t**) MoCA (all entries).

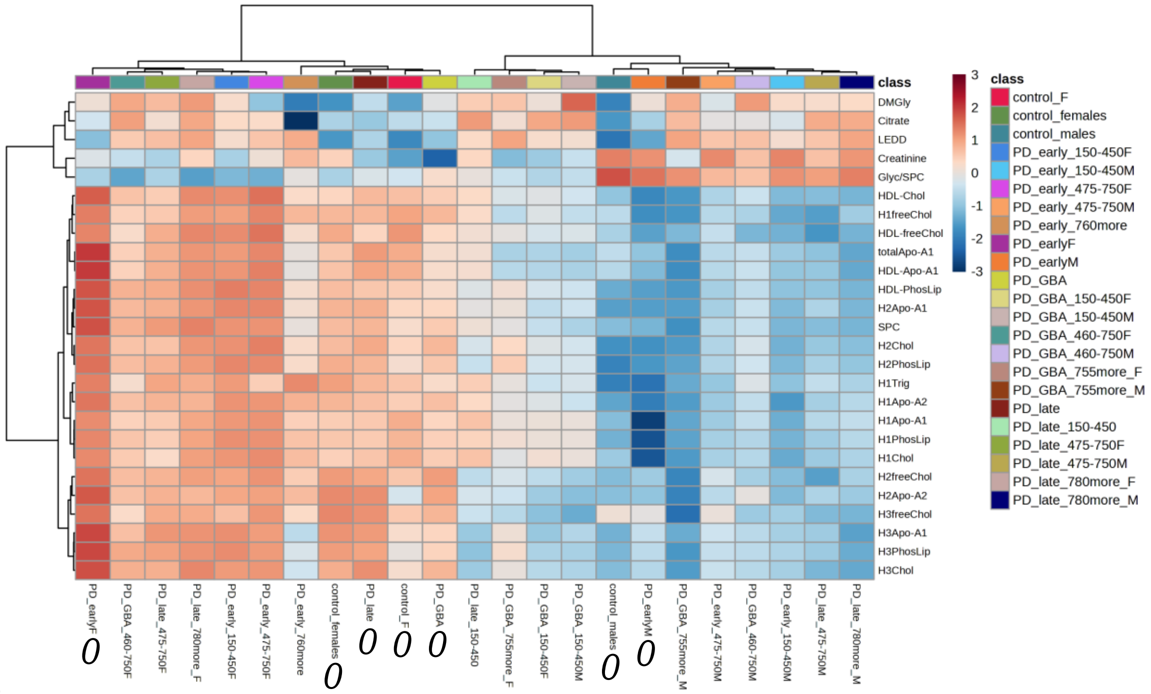
class
DMGly
Citrate
LEDD
Creatinine
Glyc/SPC
HDL-Chol
H1freeChol
HDL-freeChol
totalApo-A1
HDL-Apo-A1
HDL-PhosLip
H2Apo-A1
SPC
H2Chol
H2PhosLip
H1Trig
H1Apo-A2
H1Apo-A1
H1PhosLip
H1Chol
H2freeChol
H2Apo-A2
H3freeChol
H3Apo-A1
H3PhosLip
H3Chol
PD_earlyF
PD_GBA_460-750F
PD_late_475-750F
PD_late_780more_F
PD_early_150-450F
PD_early_475-750F
PD_early_760more
control_females
PD_late
control_F
PD_GBA
PD_late_150-450
PD_GBA_755more_F
PD_GBA_150-450F
PD_GBA_150-450M
control_males
PD_earlyM
PD_GBA_755more_M
PD_early_475-750M
PD_GBA_460-750M
PD_early_150-450M
PD_late_475-750M
PD_late_780more_M
3
2
1
0
-1
-2
-3
class
control_F
control_females
control_males
PD_early_150-450F
PD_early_150-450M
PD_early_475-750F
PD_early_475-750M
PD_early_760more
PD_earlyF
PD_earlyM
PD_GBA
PD_GBA_150-450F
PD_GBA_150-450M
PD_GBA_460-750F
PD_GBA_460-750M
PD_GBA_755more_F
PD_GBA_755more_M
PD_late
PD_late_150-450
PD_late_475-750F
PD_late_475-750M
PD_late_780more_F
PD_late_780more_M

A)

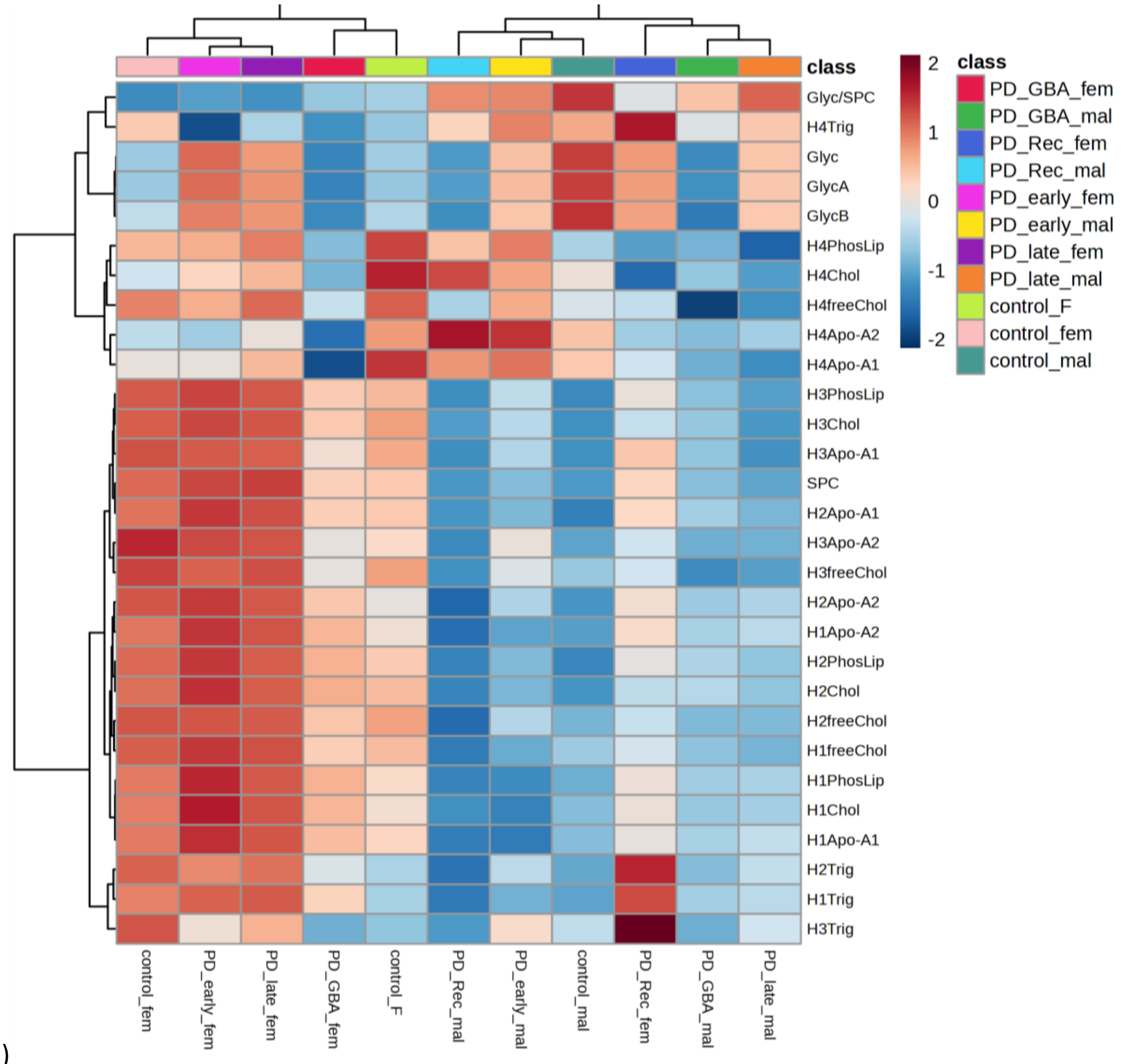
class
Glyc/SPC
H4Trig
Glyc
GlycA
GlycB
H4PhosLip
H4Chol
H4freeChol
H4Apo-A2
H4Apo-A1
H3PhosLip
H3Chol
H3Apo-A1
SPC
H2Apo-A1
H3Apo-A2
H3freeChol
H2Apo-A2
H1Apo-A2
H2PhosLip
H2Chol
H2freeChol
H1freeChol
H1PhosLip
H1Chol
H1Apo-A1
H2Trig
H1Trig
H3Trig
control_fem
PD_early_fem
PD_late_fem
PD_GBA_fem
control_F
PD_Rec_mal
PD_early_mal
control_mal
PD_Rec_fem
PD_GBA_mal
PD_late_mal
2
1
0
-1
-2
class
PD_GBA_fem
PD_GBA_mal
PD_Rec_fem
PD_Rec_mal
PD_early_fem
PD_early_mal
PD_late_fem
PD_late_mal
control_F
control_fem
control_mal

B)

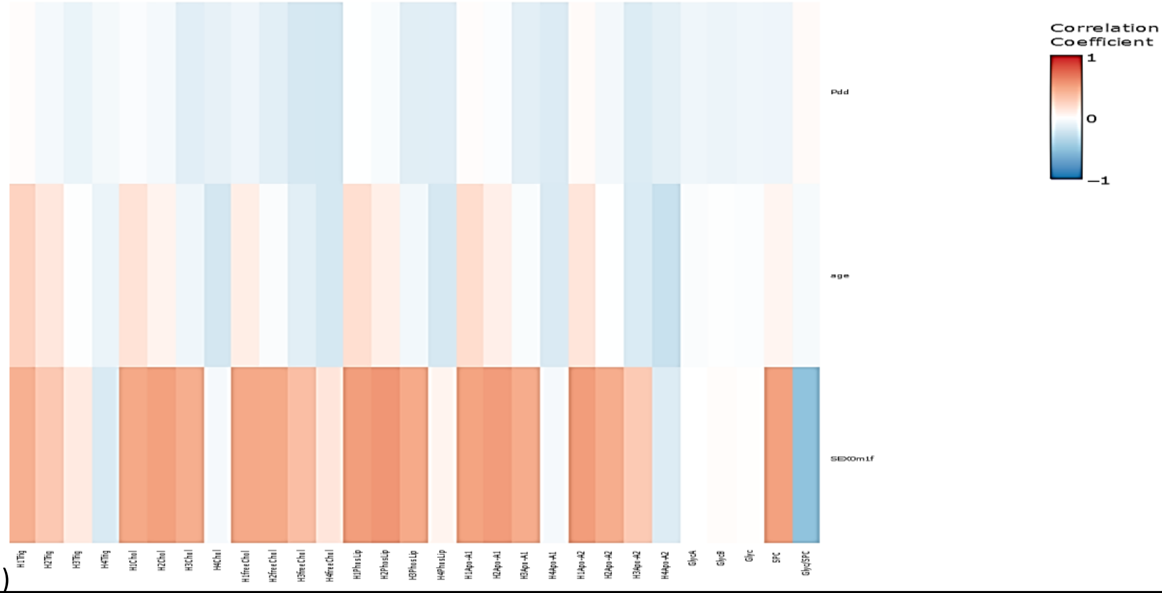

C)

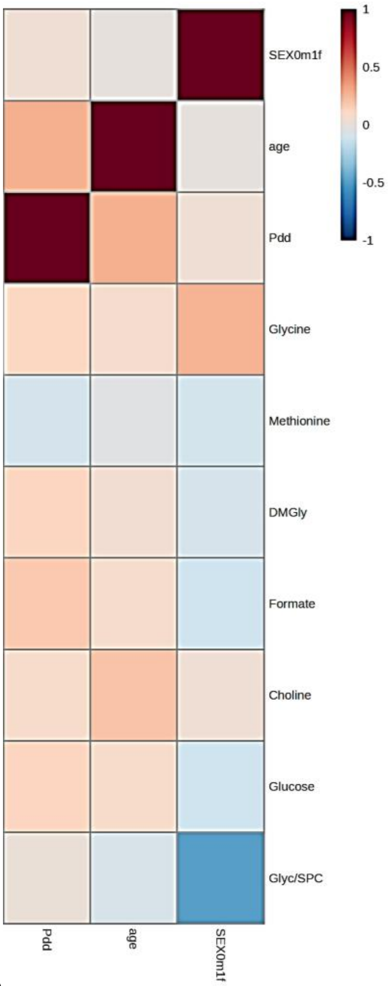

D)

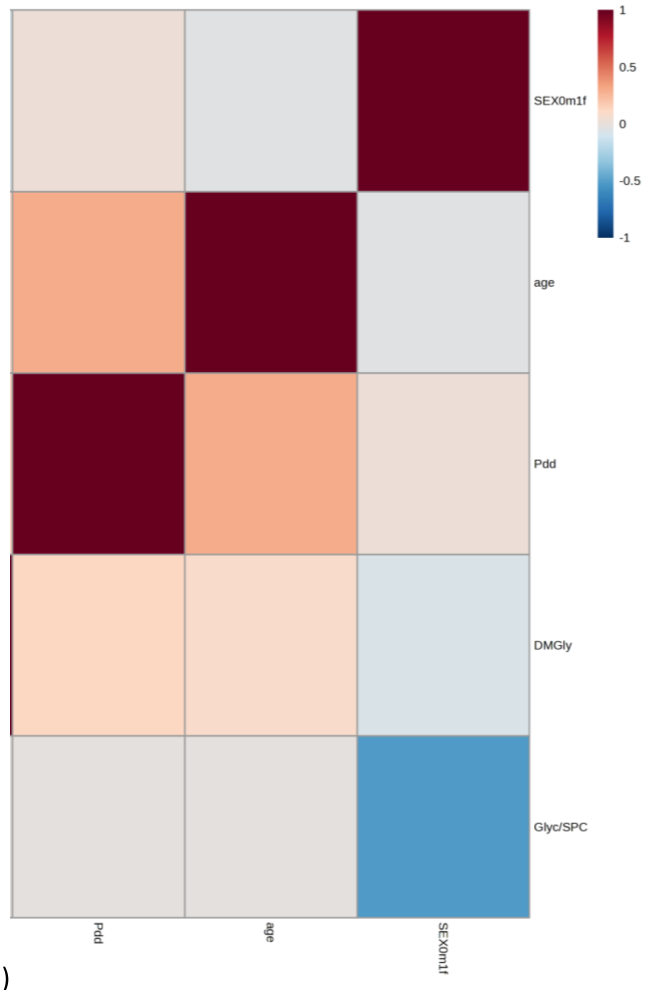

E)

Supplementary Figure 4: Correlation and Heatmap plots.

(a) Sorted PD patients (averaged) into groups based on how much L-Dopa they take each day to see if there are any links between metabolic differences and drug treatment.

(b) & (c) Correlation analysis and Heatmap plots of the HDL subfractions of lipoproteins and the inflammatory markers (Glyc; SPC) in males and females to see how inflammation and metabolic

changes are connected.

(d) An integrated (correlation) analysis of blood methionine, DMGly and formate levels to check for any abnormalities in one-carbon metabolism in people with PD. Additional parameters were included.

(e) Correlation analysis in search for a connection between DMGly and LEDD in both GBA mutation positive and sporadic PD groups to work out if DMGly changes can be affected by dopaminergic treatment.

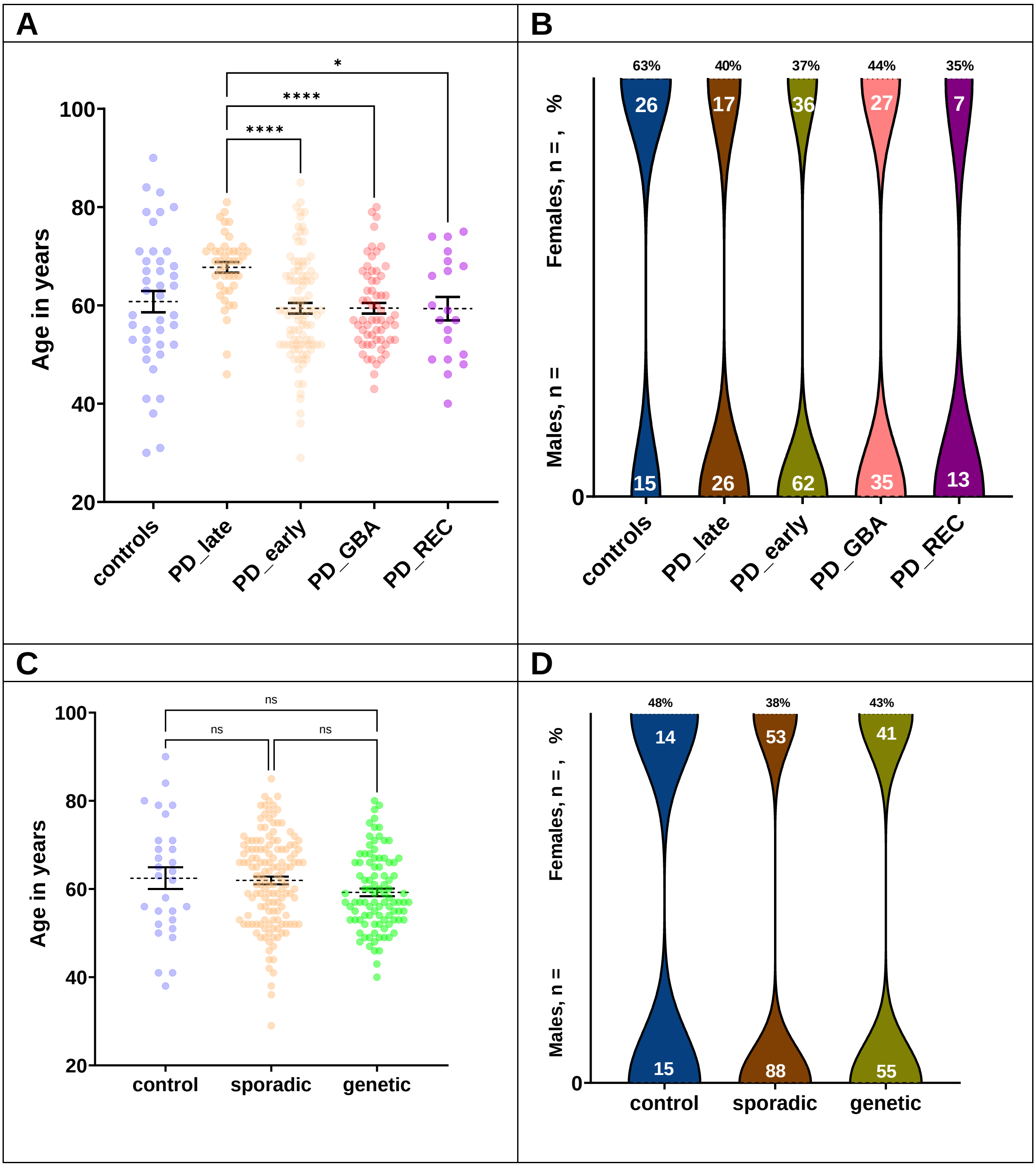

Supplementary Figure 5: Unchanged controls (by sex) in age group distribution closer and further group characteristics onto sporadic and genetic super groups. (**a**) Describtional (mean ± SEM format) statistics plot on age, whereas the old (all subject samples) of the control groups patients were utilized; (**b**) Describtional statistics on sex, in which a balance corridor of 40% ±10% among groups was not reached. (**c**) Describtional (mean ± SEM format) statistics plot on age, whereas the groups of controls, sporadic, and genetic were used; (**d**) Describtional statistics on sex for the given three groups, in which a balance corridor of 40% ±10% among groups is observed.
